# The Elusive “Switch Process” in Bipolar Disorder and Photoperiodism: A Hypothesis Centering on NADPH Oxidase-Generated Reactive Oxygen Species Within the Bed Nucleus of the Stria Terminalis

**DOI:** 10.3389/fpsyt.2022.847584

**Published:** 2022-06-16

**Authors:** Martin N. Raitiere

**Affiliations:** Department of Psychiatry, Providence St. Vincent Medical Center, Portland, OR, United States

**Keywords:** bipolar disorder, photoperiodism, seasonal affective disorder, bed nucleus of stria terminalis, NADPH oxidase, reactive oxygen species, astrocytes, dopamine A10dc neurons

## Abstract

One of the most striking and least understood aspects of mood disorders involves the “switch process” which drives the dramatic state changes characteristic of bipolar disorder. In this paper we explore the bipolar switch mechanism as deeply grounded in forms of seasonal switching (for example, from summer to winter phenotypes) displayed by many mammalian species. Thus we develop a new and unifying hypothesis that involves four specific claims, all converging to demonstrate a deeper affinity between the bipolar switch process and the light-sensitive (photoperiodic) nonhuman switch sequence than has been appreciated. First, we suggest that rapid eye movement (REM) sleep in both human and nonhuman plays a key role in probing for those seasonal changes in length of day that trigger the organism's characteristic involutional response (in certain animals, hibernation) to shorter days. Second, we claim that this general mammalian response requires the integrity of a neural circuit centering on the anterior bed nucleus of the stria terminalis. Third, we propose that a key molecular mediator of the switch process in both nonhumans and seasonal humans involves reactive oxygen species (ROS) of a particular provenance, namely those created by the enzyme NADPH oxidase (NOX). This position diverges from one currently prominent among students of bipolar disorder. In that tradition, the fact that patients afflicted with bipolar-spectrum disorders display indices of oxidative damage is marshaled to support the conclusion that ROS, escaping adventitiously from mitochondria, have a near-exclusive pathological role. Instead, we believe that ROS, originating instead in membrane-affiliated NOX enzymes upstream from mitochondria, take part in an eminently physiological signaling process at work to some degree in all mammals. Fourth and finally, we speculate that the diversion of ROS from that purposeful, genetically rooted seasonal switching task into the domain of human pathology represents a surprisingly recent phenomenon. It is one instigated mainly by anthropogenic modifications of the environment, especially “light pollution.”

## Introduction

The elucidation of the mechanism subserving the remarkable phenomenon whereby patients with bipolar affective disorders (BDs) switch rapidly from one mood state to another has been identified, perhaps with forgivable hyperbole, as the “holy grail” of research in bipolar disorders (1, 2). The hypothesis presented here proposes a mechanism for the “switch process” in question. It does so by developing the physiological kinship between the pervasive pattern of seasonal modulation displayed by many species of nonhuman life and the seasonal configuration that obtains in a significant subset of patients with bipolar mood disorders (3). [Such a configuration is displayed by definition in seasonal affective disorder [SAD] (4), an entity that overlaps strongly with BD type II [BDII] (5)]. As is well known, many animal species orient themselves to the sidereal calendar by tracking the length of day or, to put it differently, the gradually changing ratio between the illuminated (photophase) vs. the dark (scotophase) portions of the day^1^; accordingly such a capacity is termed photoperiodic. Upon encountering a series of changing day lengths signaling the approach of the most hazardous season (generally winter), such species undertake a gradual phenotypic “switch” of their own eventuating in an involutional state contemporaneous with that season, thus maximizing their chance for survival. We will propose that these two seasonal processes, nonhuman and human, mutually enlighten each other—and indeed that their core switch mechanisms will prove to be virtually identical. Having defended that basic identity, we shall be required to explain how the pathological human condition arose out of a strongly conserved and entirely nonpathological photoperiodic ancestry.

Box 1 **Glossary of selected terms.**

Aerobic Glycolysis (AG): a biochemical cascade that converts glucose into lactate through 10 consecutive enzymatic reactions. In the CNS AG is carried out largely by astrocytes. The qualifier aerobic means that the cascade can begin and run to completion in the presence of oxygen—something that would ordinarily cause the cell to metabolize glucose through another pathway called oxidative phosphorylation.

Crepuscule: strictly speaking, like twilight, this may refer to either dawn or dusk. In the current paper, it indicates the particular twilight time that coincides with the animal's transition from sleep to waking. This varies as a function of the mammal's activity pattern: for nocturnal species, it means dusk; for diurnal ones, dawn.

Crepuscule Change Detector: a homegrown term for a CNS mechanism that is tuned to distinguish day-over-day luminance changes in the pre-wake crepuscule, particularly in autumn as the days are getting shorter. This mechanism may involve a comparator sensitive to any difference between yesterday's temporal activity pattern (as reflected in the comparatively inertial circadian activity rhythm) vs. today's luminance signal as sampled during the relevant crepuscule. (The sampling process itself takes place in REM sleep.) We tentatively locate such a comparator in the DMN of the hypothalamus.Hormesis: the concept according to which a minimal dose of an otherwise noxious substance or influence calls forth an answering response the beneficial effects of which clearly outweigh the potentially harmful effects of the initial or triggering dose.

Incremental Augmenter: homegrown term for a subtle CNS device that iteratively amplifies the animal's SD response from day to day, doing so by transmitting the seasonal decision formulated within the suprapontine seasonal module to the brainstem (pontine) REM sleep generator. Such a daily effect probably involves augmentation of REM density (see below).

LD or LDs: long or longer days, sometimes casually identified with those of summer. Here it has the comparative meaning *longer*, i.e., that portion of the calendar in which photophase is longer (and scotophase shorter) than it was on the previous day. This obtains not in astronomically defined summer but from the winter solstice to the summer solstice.

NADPH Oxidase (NOX): a family of enzymes responsible for generating ROS. In our hypothesis, one or more of these enzymes generates the ROS that mediate the switch from the first (hyperarousal) to the second (hypoarousal) phase of the mammal's response to SDs.

Photophase: the illuminated portion of the daily cycle.

Reactive Oxygen Species (ROS): a group of highly reactive compounds deriving from oxygen. Once believed to have a largely destructive role, these have in recent years been shown to carry out physiological assignments, functioning for example within signal cascades.

REM Density: a measure of that portion of the REM sleep phase that is occupied by the characteristic rapid eye movements (REMs) after which this phase in general is named. It may be calculated directly as a percentage; an alternative, somewhat indirect, measure involves assaying the duration of individual bouts of REMs. A REM sleep phase may last 60-90 minutes in human beings; a bout of the characteristic eye movements may last from seconds to over two minutes. Greater REM density of biological significance generally means longer bouts of individual REM episodes (i.e., lasting over 2 minutes), not a greater number of short REM bouts (i.e., lasting a few seconds).

Scotophase: the dark portion of the daily cycle.

SD or SDs: short or shorter days, sometimes casually identified with those of autumn and winter. Here it has the comparative meaning *shorter*, i.e., that portion of the calendar in which photophase is shorter (and scotophase longer) than it was on the previous day. This is the case not in astronomically defined winter but from the summer solstice to the winter solstice.

Seasonal module: this term is utilized here to indicate a variably (loosely or tightly) linked network of subcortical nodes that, working together, mediates the seasonal animal's response to the SDs of autumn. See Known and Likely Nodes in the Photoperiodic Network of text for a rough consensus concerning the neural nodes to be included within this module. Supra-2-Minute Interval: a homegrown term for a period of time, generally lasting between 2 and 4 minutes, that has either a sidereal or an organismal context or both together. The sidereal context as utilized here indicates the day-over-day *increase* in scotophase that transpires during a significant continuous portion of SDs. This introduces the organismal meaning: seasonal animals construe an interval *of the specified length* as license to run the AG cascade to completion. Since the sidereal supra-2-minute interval obtains roughly from August through December, AG within the animal's seasonal module (see above) generally runs to completion only within those months.

Note: acronyms or other terms not explained here, generally involving anatomic regions, are clarified within the text proper.

Thus this paper has two goals. In pursuing the first, the development of the case for a generalized mammalian switch process, we assign a significant role to reactive oxygen species (ROS) of a particular provenance, namely NADPH oxidase (NOX) enzymes. Insofar as making such a claim, this position differs from one that currently enjoys favor in bipolar research: this casts ROS, viewed as stemming primarily from stressed mitochondria, as agents of damage (6–13). Without contesting the robust evidence for oxidative harm in the bipolar patient (6–13), we locate the origin of the ROS in question in a source upstream from mitochondria, i.e., the cell membrane-anchored NOX system, and in a *nonpathological* process involving the seasonal switch mechanism. Thus our second goal: to adjudicate between these two competing ROS-focused bipolar hypotheses.

The two ROS hypotheses need not, of course, be mutually exclusive. Indeed we arrive at a perspective which, accommodating both, allows each to hold sway at different phases of mammalian evolution. In the first and by much the longest phase, mammals exercised a “pure” photoperiodic strategy including, we believe, the ROS-based switch mechanism outlined here. It is possible that elements of this strategy carried, with modifications, into certain early hominin species (14). Yet two factors intrinsic to human evolution gradually complicated this picture. As hominins gradually migrated from the equator into temperate zones, selection pressures likely further mitigated their photoperiodic heritage so as to allow them to deal actively, not passively, with winter. Secondly, and quite recently within the Anthropocene, a different and entirely unprecedented challenge to photoperiodism has arisen: anatomically modern human beings have come to modify their own environment by generating “light pollution.” That is, through artificial light they have degraded the remarkable step-off between the lighted vs. the dark phases of the sidereal cycle upon which photoperiodism depends. This change has given admission, we shall propose, to ROS working as a damage agent rather than a player in a signal cascade.

Thus modern human beings with BDII/SAD may represent a complex amalgam featuring both constructive and destructive effects of luminance and ROS. To offer an analogy from another medical domain, the patient with congestive heart failure may exhibit symptoms through which one may detect underlying physiology: while the enlarged heart denotes a clearly pathological feature, the avid renal tubular reabsorption of sodium points to an aspect of physiology that is working only too well. Similarly, the BD patient who develops mania in autumn or spring may display delusions of grandiosity that are obviously pathological—and yet the timing of his episode will represent an atavistic remnant of his photoperiodic physiology.

**Figure 1:**
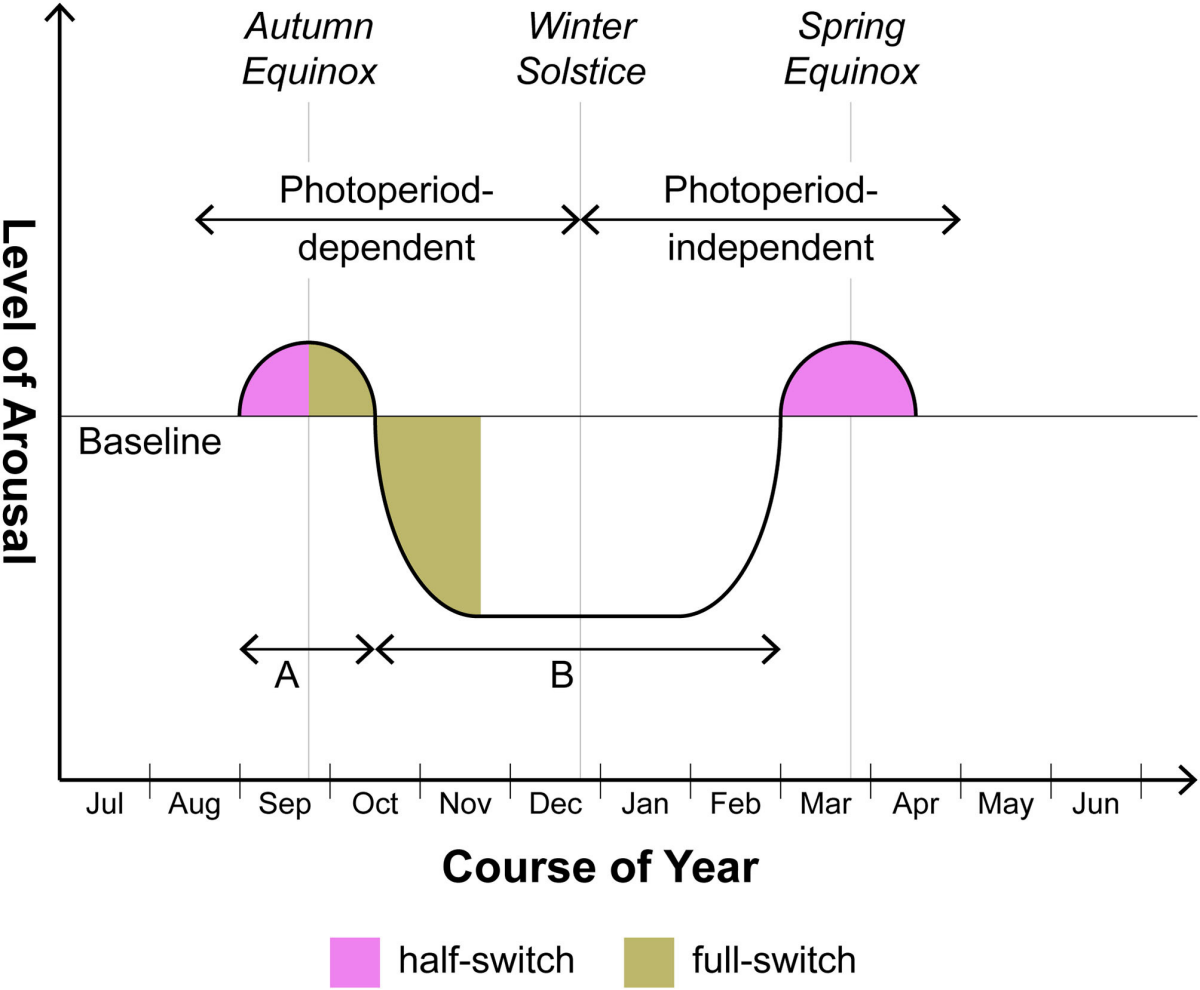
Seasonal switching in photoperiodic mammals including humans with BDII/SAD. This is a schematic rendition of the manner in which arousal varies seasonally above and below baseline over 12 months. Note that the supra-baseline or hyperarousal phase generally does not reach the amplitude or the duration characteristic of the infra-baseline or hypoarousal phase. In human beings, the degree of amplitude of supra-baseline arousal comprises a major differentiating feature of BDI (hyperarousal of greater amplitude, i.e. mania) vs. BDII (hyperarousal of lower amplitude, i.e., hypomania). Although focusing with respect to humans on BDII/SAD, this schematic does not exclude the BDI patient who in certain cases may also merit the seasonal specifier. In those cases, the amplitudes of episodes above and below baseline will be roughly equal but the timing of episodes will still center on the equinoxes. As to duration, the ratio of time spent in hyperarousal (time A) vs. hypoarousal (time B) may prove to be roughly the same in both nonhuman and human photoperiodic substitute organisms for animals (on the order of 1:5-1:8). However animals may separate from humans in various ways. As discussed in the text, the autumnal hyperarousal episode in smaller nonhuman species frequently requires controlled omission of temperature change to be unmasked. With larger nonhuman quadrupeds, the hyperarousal phase (= the rut), while fairly obvious, is less likely to fall precisely at one of the equinoxes in comparison to smaller nonhuman species or human beings: timing of gestation so as to ensure parturition at the safest period of the year trumps precise fidelity to the equinox.

Now it is mandatory to consider whether some sort of logical fallacy is involved in the application of the clinical terms mania and depression to nonhuman mammals. A possibly less contentious dimension with application both to nonhuman animals and human beings with SAD/BDII is *arousal*. Thus in Figure 1, a schematic diagram applying to seasonal organisms whether nonhuman or human, the baseline depicted represents the degree of arousal under non-stressed conditions; even the Syrian hamster makes seasonal excursions above and below that reference point. While the excursion into states of *hypoarousal* (*i.e.*, torpor and hibernation) is well known and in fact is generally viewed as coterminous with the SD response, a preceding excursion into *hyperarousal* has only recently attracted attention. The Syrian hamster, for example, does not descend quietly into torpor over the months-long course of SDs. The hyperarousal phase sometimes requires artificial “editing” in the laboratory of the autumn-winter environment, i.e., a partial representation of that season including SDs but excluding a drop in temperature, for prolonged and patent expression: thus if the photoperiod is switched from LDs to SDs but temperature is kept unchanged, the hamster will enter a phase of *increased* aggressiveness (15–18). Clearly such organisms are hyperaroused. This behavioral phase may continue indefinitely unless decrease of ambient temperature is added to extended scotophase; only thereafter does the animal progress into torpor (19). In our view the hyperarousal phase should not be dismissed as a laboratory artifact; what is artificial is rather *prolongation* of this phase beyond its relatively brief naturalistic life (see below). In any event we do not lack other and more manifest examples of seasonal hyperarousal in vertebrates with some kinship to mania: these include, in larger quadrupeds, the constellation of irritability, hypersexuality, and peak reproductive potency comprising the rut (20) and, in avian species, two remarkable phenomena associated with migration, namely *Zugunruhe* or the migratory-epoch restlessness of caged birds as well as the profound intra-migratory insomnia which has been proposed as a model for manic insomnia (21). In both the smaller nonhuman and the human (BDII/SAD) cases, such episodes of hyperarousal frequently obtain in the weeks flanking the autumn and spring equinoxes whereas the intervening involutional period is more extended, occupying much of autumn and winter. At least in smaller animals, then, the phase involving hyperarousal may be overlooked.

By way of simplifying matters, we shall focus on the response of mammals at large—including human beings with BDII/SAD—to the shorter days (SDs) of autumn rather than to the longer days (LDs) of spring. We do so for two reasons. First, although a relevant study drawing on 51 papers, many retrospective and hospital-based, found that manic episodes develop to a lesser extent in autumn than in spring/summer (22), one carefully executed prospective clinical study involving BD I and II patients discovered a highly significant peak in manic symptoms in the weeks surrounding the autumn equinox, a pattern that was most pronounced in BDII individuals (23). Second, in many photoperiodic species the winter→ spring recovery of baseline functionality, which would seem at first glance to represent the mere obverse of the autumn→ winter descent into involution, betrays an additional and still poorly understood layer of complexity. For classic seasonal mammals such as the Syrian hamster begin to recover sexual and reproductive potency in late winter not because they are responding to the LDs then extant but rather because they have become refractory, after some 20 weeks, to SD signals. Thus they gradually regain their baseline functionality *in phase with, but not as a result of*, those LDs (24). Dodging the still largely opaque problem of emergent refractoriness to SDs allows us to attend at some length to the *direct* pro-involutional effects of autumnal SDs upon mammalian physiology—a field offering its own generous quotient of unresolved issues. We shall have work enough to do addressing those.

## Recent Advances and Some Outstanding Unresolved Issues in Photoperiodism With Proposed Solutions

### Known and Likely Nodes in the Photoperiodic Network

After the discovery that melatonin functions as key hormonal proxy for scotophase (25), attention shifted to the brain regions capable of decoding or “readout” of the melatonin signal. That research program has consistently identified the dorsomedial nucleus (DMN) of the hypothalamus as a necessary node in readout of the SD message (insofar as involving melatonin) (26–29). The DMN has an established influence upon two major modulators of arousal, the autonomic nervous system (ANS), especially its sympathetic arm, and the hypothalamic-pituitary-adrenal (HPA) axis (30); we shall attend to its seasonal adjustment of arousal through both of these systems. Other nodes attested by lesioning to belong within the mammalian seasonal network include the ventromedial nucleus (VMN) of the hypothalamus (31) and the bed nucleus of the stria terminalis (BNST) (32) the anterior division of which has a major role in our hypothesis. Much evidence also implicates in seasonal function the orexinergic neuronal system located primarily in the lateral hypothalamic area (LHA) with a smaller contingent in the DMN (33–35).

**Figure 2:**
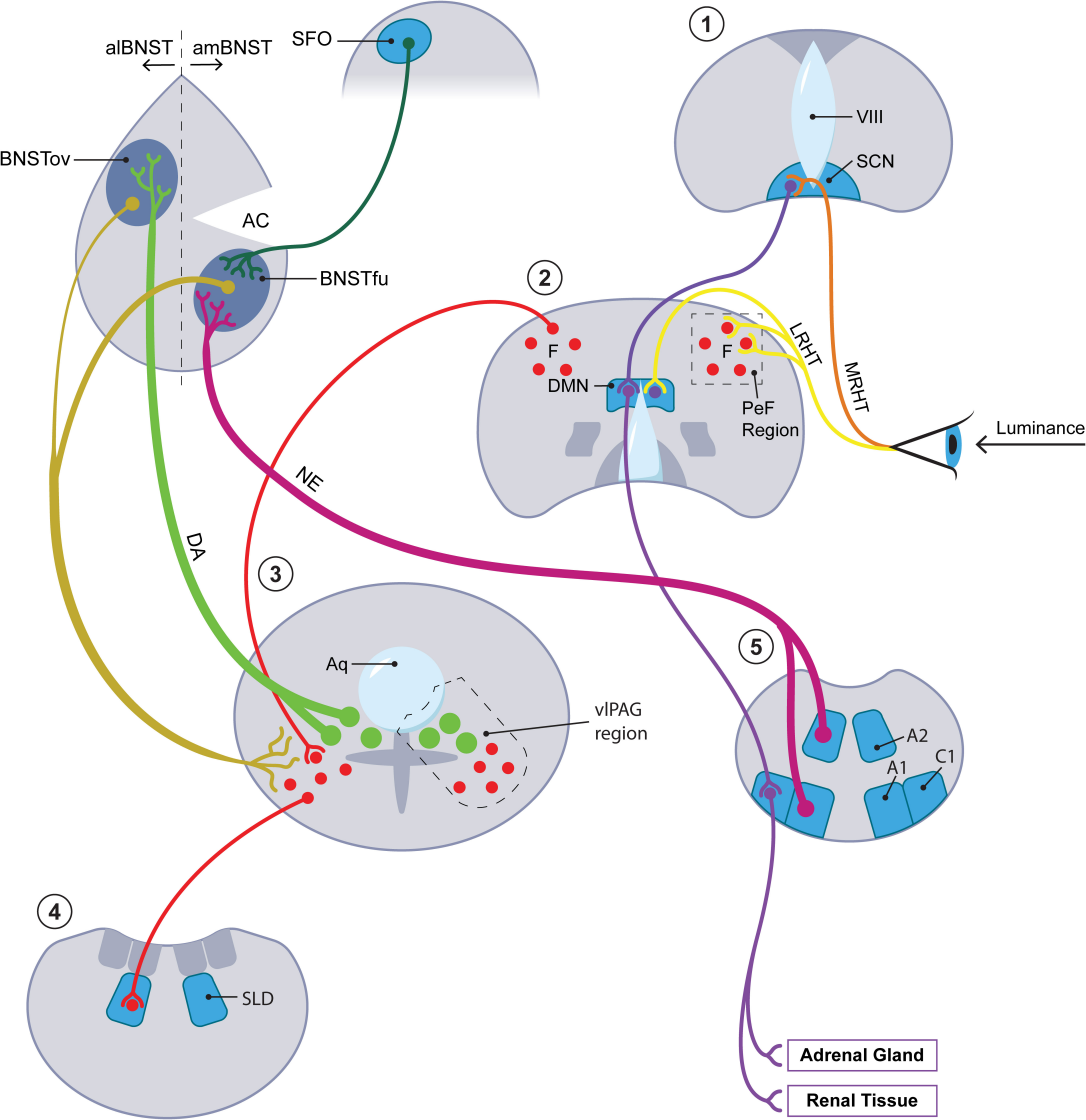
The photoperiodic network. The diagonal axis going from lower left to upper right features four coronal sections with the most rostral stationed at the upper right. A fifth, most caudal in the series, is placed at the lower right of the figure. Each of the five coronal sections is numbered with a red numeral (from rostral to caudal): 1 = hypothalamus at level of SCN (for abbreviations, see below); 2 = hypothalamus at level of DMN; 3 = periaqueductal gray region at level of DA A10dc neurons; 4 = pons at level of SLD; 5 = medulla at A1/C1 level. At level 3, closed green circles represent DA A10dc neurons; closed red circles, GABA neurons. At upper left, the diagram concentrates upon the aBNST but also includes, as representative of all of the circumventricular organs, the SFO. Key anatomic authorities are given in text. Terminology of BNST subnuclei generally derives from Swanson and colleagues [here, references (161, 162)]. We do not have room here or in the text for an extended discussion of the multiple cases of anatomic reciprocity joining suprapontine and pontomedullary sites. In brief, key arousal-related monoamines with cell bodies at pontomedullary levels, especially the DA neurons in the vlPAG at level 3 and the medullary NE neurons at level 5, project rostrally to suprapontine sites, especially the hypothalamic levels 1 and 2 and the aBNST. (To minimize clutter, afferents from below involving DA and NE to suprapontine regions are given for the aBNST but omitted for the hypothalamus.) Primarily at the suprapontine hypothalamic sites, the arousal-inflected monoamine systems mesh with afferents (especially those traveling in the LHRT) bearing luminance data from the retina. Neurons within the suprapontine sites then project back into the vicinity of the pontomedullary sites from which the rostrally projecting tracts originated. In brief, the reciprocating circuits of interest to us move from arousal region→ luminance modulation→ arousal region. This paradigm holds most clearly for intersections with luminance data which take place in hypothalamic section 2. While the paradigm in question may seem to obtain less clearly for the circuit traveling through the aBNST, it does in fact hold for this region once important connections between the aBNST and the DMN/PeF [not shown here; see Hahn and Swanson (161)] as well as between the aBNST and the brainstem [see Dong et al. (162)] are factored in. Only the most critical aBNST-related pathways are rendered here. See especially the gold-coded descending tracts running from both the al- and the amBNST into the vlPAG. In summary, the aBNST satisfies criteria for a reciprocating circuit which may be said to capture luminance information for arousal and for sleep/wake circuitry. IIIV, 3^rd^ ventricle; A1, A1 medullary cell group; A2, A2 medullary cell group; AC, anterior commissure; alBNST, anterolateral division of BNST; amBNST, anteromedial division of BNST; Aq, cerebral aqueduct; BNST, bed nucleus of stria terminalis; BNSTfu, fusiform subnucleus of BNST; BNSTov, oval subnucleus of BNST; C1, C1 medullary cell cluster; DA, dopamine; DMN, dorsomedial nucleus of hypothalamus; LRHT, lateral RHT; MRHT, medial RHT; NE, norepinephrine; PeF, perifornical region of lateral hypothalamus; RHT, retinohypothalamic tract; SCN, suprachiasmatic nucleus of hypothalamus; SFO, subfornical organ; SLD, sublaterodorsal nucleus; vlPAG, ventrolateral periaqueductal grey.

Other kinds of evidence suggest that tissues within or directly adjoining the brain's ventricular system participate in the mammalian SD response. Different members of the circumventricular organ (CVO) group along with the closely related pineal gland and choroid plexus not only secrete melatonin (the pineal gland) but also transmit the effects of leptin, a hormone with significant ties to seasonality (36), to the sympathetic arm of the ANS [the subfornical organ [SFO] (37)] and generate seasonally appropriate neurotrophic factors [the subcommissural organ [SCO] and the choroid plexus (38)]. Relevant in this connection is the discovery in 2012 by Nedergaard's group of what they named the glymphatic system (39, 40). Before that discovery, strong seasonal variation had been documented in the brain's transport of pertinent molecular mediators through cerebrospinal fluid (CSF) (41). Although to our knowledge investigators have not explored the seasonal ramifications of the glymphatic system, we levy that concept here to strengthen several themes which we believe relevant to photoperiodism. First, the glymphatic concept sanctions the centrality within our hypothesis of astrocytes and the gliotransmitters they produce including, as we shall see, ATP and lactate. Second, we are encouraged by the glymphatic template to blur the boundaries between the strictly circumventricular organs and other periventricular tissues that we envision as components of the photoperiodic network. These periventricular sites include not only established seasonal players such as the DMN but also other loci such as the ventrolateral periaqueductal gray (vlPAG) and the oval subnucleus of the BNST; the latter two comprise the locations respectively of the cell bodies and the main terminal field of the dopamine (DA) A10dc cell system. Thus we will not draw any strict distinction as regards seasonal function between the circumventricular organs *per se* and these periventricular tissues or, accordingly, between CSF and interstitial fluid (ISF). Third, the notion that glymphatic flow actively distributes astrocytic products through CSF/ISF primarily during sleep hovers in the background of our claim that the mammal carries out a crucial fluid transport component of its SD response while sleeping (though we have in mind a more differentiated portion of sleep than has obtained thus far in discussions of the glymphatic system). Figure 2 provides a simplified schematic of the photoperiodic network including the CVOs.

### The Supra-2-Min Problem

The onerous nature of approximating in the laboratory the extremely slow change of naturalistic photoperiod may have led to some unfortunate shortcuts. Understandably, the classic laboratory protocol simplifies matters by comparing animals under two photoperiodic conditions, a fixed LD (e.g., 16 h of light: 8 h of dark [L: D 16: 8 where L and D refer to light and dark respectively]) vs. a fixed SD (e.g., L: D 8: 16) schedule. Such a procedure has proven useful for defining an absolute value for the minimum duration of scotophase that triggers involution, e.g., 11.5 h for the Syrian hamster. Yet no animal in the wild ever experienced two consecutive days (let alone more) of *identical* photoperiod. In the wild, it is the barely perceptible day-over-day *extension* of scotophase in late summer and autumn that must somehow be “read” by the organism and must then be integrated over a number of days. [This is compatible with the view that a series of such days, perhaps as few as 7–14, is required for the secure fixing or “memorizing” of that sensory decision (42)]. Since for temperate-zone mammals we have good warrant for the emergence in some mammals of photoperiod-driven neuroendocrine change in August (43), i.e., a month at the beginning of which scotophase is increasing by about 2 min daily, and since at such latitudes that daily extension of scotophase rarely reaches 4 min at its late-September maximum (44), we can safely assume that most *effective* values for day-over-day scotophase change fall on a spectrum between 2 and 4 min. For simplicity we shall refer hereafter to the *effective* SD signal as one with a “supra-2-min” value. This will be short for: a rate of expansion of day-over-day scotophase between an approximate minimum of 2 and an approximate maximum of 4 min.

As demonstrated by resonance or Nanda-Hamner protocols, the animal reads environmental luminance signals most efficiently when they recur with 24-h periodicity (45). This has long implied that the seasonal animal may be sensitive to such signals within a given segment of its own endogenous rhythmicity. But the definition of this segment has proven surprisingly problematic. For example, attempts were made to construe sensitivity to the slow change of photoperiod on the model of classic work establishing circadian response curves to brief pulses of light administered to animals in constant darkness and yielding *immediate* phase shifts. These attempts were disappointing insofar as such pulses inevitably impaired or aborted, rather than potentiating, the involutional response (46). Evidently there is some difference between an animal's handling of what may be termed arousing emergencies [whether involving light pulses or so-called nonphotic stimuli (47)] that *immediately* reset the circadian activity rhythm vs. its negotiating the slow expansion of scotophase that takes place in SD insidiously over weeks and that is not accompanied by such resetting. Perhaps there is something amiss with the frequently unspoken assumption that the animal's “endogenous” rhythmicity serves as a baseline upon which varying “exogenous” photoperiods impinge. What if the seasonal animal, rather than being *passively* subjected to the external influence of luminance change, were constantly *searching* for evidence of that change? And what if the *search*, not the mere reception of the sensory signal, transpired within a segment of its sleep cycle that was adapted for maximal sensitivity to a crepuscular grade of luminance?

**Figure 3:**
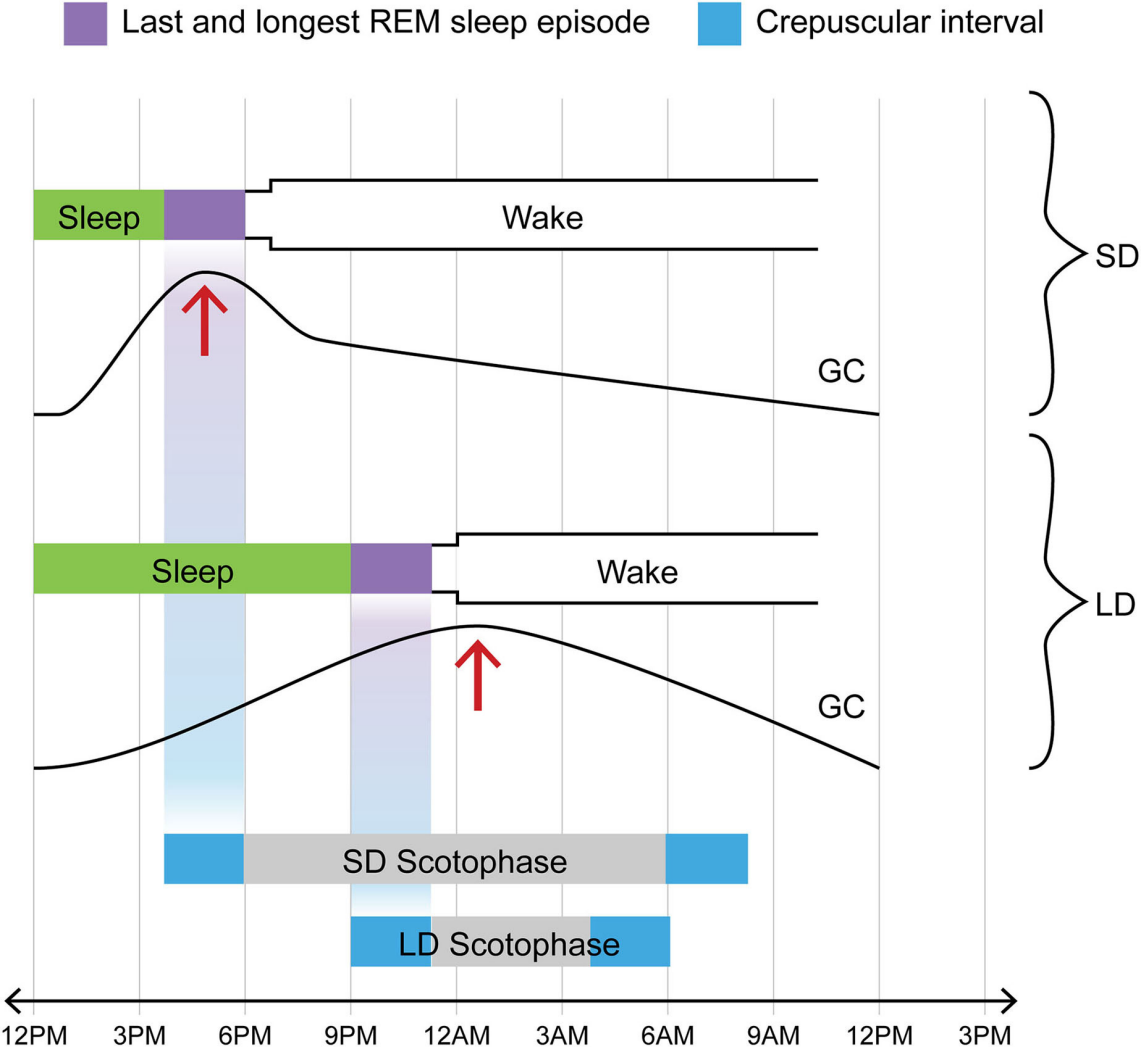
One of two crepuscular intervals coincides temporally with final REM sleep episode in any photoperiod; in contrast, glucocorticoid pulse profile varies with photoperiod. Note that *for both LD and SD*, the L→ D crepuscular phase (in this diagram illustrating the case for a nocturnal animal, the bright blue rectangle at the *left* extremity of the bar marking scotophase) and the final REM sleep episode (purple rectangle immediately preceding shift to waking) are positioned on the same vertical axis, i.e., they invariably track together in time in time regardless of photoperiod. Exactly the same temporal coincidence would hold for a diurnal species. In the diurnal case, however, the crepuscular phase in lockstep with the final REM sleep episode would involve the D→ L transition (shown here by the bright blue rectangle at the *right* extremity of the bar marking scotophase). Now in contrast to the temporal coincidence between one of the two crepuscular transitions and the sleep-wake transition, one that holds in all photoperiods, consider the case of the GC pulse (addressed in text). The acrophase of that daily pulse profile is differentially affected by LD vs. SD: it is shifted to an earlier position (phase-advanced) in SD but not in LD. The text addresses the likely cause of this shift, namely the sympathetic activation which surfaces at around 2 weeks of SD (see IIB and IVB for relevant citations). Such a phase-shift causes the GC acrophase in SD to be attracted into the concluding REM sleep interval. In part through augmented calcium signaling, the elevated GC titer contributes within that interval of sleep to the generation of neurotrophic or prosurvival agents including BDNF and FGF. This is, in our view, the case for DA and medullary NE neurons affiliated with the aBNST. It does not necessarily hold for a radically different neural circuitry, that involving the hippocampus and cortex and afferented by the LC NE system.

In 3 papers published in 1978-1988, one group found evidence of such a possibility (48–50). The epochs of rapid eye movement (REM) sleep or, more precisely, of REMs themselves, the phasic oculomotor events after which the entirety of the sleep phase in question is named, were noted to occasion radically augmented sensitivity to a certain species of luminance-related information. Thus sectioning the extraocular muscles of rats deprived them of the ability to adjust their circadian activity rhythm to the light: dark cycle (49). Now the gradual “homeostatic” strengthening of REMs over the course of sleep (51–55) brings the last, longest, and most vigorous REM sleep episode—that immediately preceding the shift from sleep to waking—into temporal register with the sidereal transition between darkness and light (whether L→ D for the nocturnal animal or D→ L for the diurnal one). This means that the animal by means of its REMs may be probing most effectively for luminance signals at precisely the temporal interval when luminance is *changing* (see Figure 3).

Evidence developed in the interval since these rather neglected papers were published strengthens the authors' hypothesis. Studies in cats have shown that one aspect of REMs involves the globes' being directed downward, nasally, and not fully convergently (56); this implies irradiation [by luminance signals detectable in sleep through closed eyelids (57, 58)] of the superior temporal retinal quadrants. These quadrants, in the Syrian hamster at any rate, harbor the very subset of intrinsically photosensitive retinal ganglion cells (ipRGCs) that projects both directly via the lateral retinohypothalamic tract (LRHT) as well as indirectly through an intermediate station in the anteroventral LHA (LHAavr) into the DMN as well as the perifornical region (PeF) of the LHA (59–63). Rapid eye movements, then, differentially irradiate segments of the retina that transmit to the hypothalamus photoperiodically salient (but not image-forming) luminance data.

Although our purpose here is not to elaborate a full model for what may be termed the “crepuscule change detector,” i.e., that device tuned to detect the day-over-day extension of darkness into the organism's last REM sleep episode, we note that a likely candidate for such a device may be the DMN itself, perhaps the best-attested photoperiodic node. For two data streams mandatory for such a device, one carrying *luminance* data via the retinofugal LHRT (see above) vs. another carrying strictly *circadian* data by means of a direct projection from the SCN (64) converge here. Also consistent with a DMN location for the crepuscule change detector would be one of the earliest signs of the SD response: namely augmented sympathetic activity, including without being limited to renal sympathetic nerve activity (RSNA). Studied over many years by T. Bartness and colleagues (65–67), this increased sympathetic activity surfaces as early as day 14 of SD exposure (65). Although many supraspinal sites within the central autonomic network (68) could participate in driving such SD-related sympathetic augmentation, the DMN may have a greater claim to primacy here than another candidate, the paraventricular nucleus (PVN) of the hypothalamus (30). In summary, a relatively simple DMN-based comparator with two inputs (bearing photic and circadian data) and one output (involving modification of sympathetic activity) will serve our purposes for now.

### How Would a REM-Based Luminance Search Device Work With the Supra-2-Min Constraint?

We have yet to explain the relationship between the proposed REM search device and the supra-2-min constraint imposed by the day-over-day expansion of autumn scotophase. Whereas length of day, year over year, is an extremely reliable (i.e., nearly noise-free) sidereal quantity (69), the biological search device involving REM sleep circuitry possesses a certain degree of built-in variability, a quality which, as we shall see, permits it to undergo seasonal modification.

One important dimension along which REM sleep exhibits relevant variability involves the magnitude or saliency of REM bursts within a given REM sleep episode. This proportion is ordinarily measured in one of two ways. One may ascertain the duration of an individual REM burst; this may have values ranging between shorter (e.g., 20 s) and longer duration (e.g., 120 s) (54). Alternatively, one may measure so-called REM density, defined as that percentage of a REM sleep period occupied by REMs; the cutoff between low and high density was placed by one group at 10%; thus any value >10% would qualify as high (53). (Either definition would serve our purpose.) It may not be fortuitous that the longer-duration REM bursts satisfy the supra-2-min constraint whereas the shorter-duration ones, missing the 2-min lower limit, do not. Given that the homeostatic aspect of REM sleep causes REM density to increase over the course of a sleep episode, the last REM episode will tend to display greater REM density than those generated earlier in sleep (51–55). *Yet we propose that even this last episode will fail to reach maximal REM density unless and until the pontogeniculooccipital (PGO) waves associated with REMs proper* (70) *summate in specified loci with luminance data carrying a SD signature*. In this case the target locus, primarily the LHA, played upon both by PGO waves (71, 72) and by the LRHT (59–63), will be excited sufficiently to trigger a neural output, here termed an *incremental augmenter*, that projects caudally back to the pontine REM sleep generator. We emphasize this caudally projecting element because it is critical for the iterative development of the SD response. Only when the incremental augmenter strengthens the REM sleep generator will it produce a series of increasingly potent high-density bursts each lasting not < 2 min. *That is, under naturalistically changing SD the number and length of such bursts within the 60–90 min of the final REM sleep episode will vary directly with length of scotophase*.

As for the location and neurotransmitter signature of the LHA system which sends a reciprocating link back to the brainstem, one likely candidate is the LHA-based melanin concentrating hormone (MCH) system. This system is not only the recipient of the PGO waves of REM sleep (71, 72) but also *disinhibits* the same sleep stage by sending MCH/GABA fibers to vlPAG GABA cell bodies that in turn project to and inhibit the pontine REM sleep generator (73–75). Thus the MCH/GABA projection is well placed to serve as the incremental augmenter required by photoperiodism. Of note, the interesting findings of increased REM sleep bout duration (74) and frequency (75) with optogenetic and chemogenetic stimulation respectively of MCH neurons were made in a non-photoperiodic context; our hypothesis would predict that monitoring MCH neurons in traditionally fixed LD vs SD would reveal increased MCH neuronal activity in the latter photoperiod. Better yet, such monitoring of animals under naturally changing photoperiods would discover that both MCH neuronal activity and REM density, reciprocally reinforcing each other, vary directly with scotophase duration.

For the notion of REM sleep as a probe for SDs, we have supporting evidence from research on patients with BD. Forty years ago, patients with a bipolar condition were found to display increased REM density compared to healthy controls (76); this has been repeatedly confirmed and elaborated (77, 78). To our knowledge, no particular extra-bipolar rationale for such increased REM density, which obtains in remitted as well as symptomatic individuals (77), has been proposed. The proper context, we believe, is provided by photoperiodism: the bipolar condition includes a heightened capacity for searching for, detecting, and responding to the autumnal extension of darkness. Note that although the BDII/SAD patient will be generating REM bursts at a density exceeding controls *throughout the year*, he or she will meet with SDs for only half of that time (and with *effective* SDs, i.e., those capable of instigating the SD response, for less than half of that time). Only beginning about August (see IIB) will his or her moderate-density REM episodes, meshing with a SD signature, become photoperiodically serviceable, i.e., disposed to progressive amplification of those bursts from moderate to high intensity.

By now the supra-2-min allowance has been shown to have a bifold meaning: it references both a *sidereal* reality, namely the decompression of scotophase in autumn, and a temporally delimited *biological* process, namely the daily quotient of the mammalian (not just the human) SD response. Much more needs to be done to unpack the latter and in particular to clarify how it exploits the sidereal constraint. To make progress on this front, we need to understand the seasonal contribution of the astrocyte.

## The Supra-2-Min Problem Revisited: Does Aerobic Glycolysis Offer a Solution?

### A Seasonal Role for AG

Let us pursue the notion that the seasonal animal can sense some relevant aspect of photoperiodic change *within a single SD sidereal cycle*. We now submit that the supra-2-min allowance gives us a clue to the nature of the molecular mediators put into play in this interval. Recall that those mediators have two tasks: they must both register the relevant change within one sidereal cycle; over a succession of cycles, they must also *amplify and fix* that change.

A still not fully understood aspect of photoperiodism, the seasonal animal's metabolic modification in autumn, may offer a relevant constellation of molecular mediators. In autumn many mammals including human beings develop noteworthy alterations in glucose metabolism, often involving a mild increase in serum glucose and multiple changes suggestive of more profoundly altered *central* glucose metabolism. [Interestingly, a small cluster of cells in the amBNST now appears to play a major role in generating a spike in glucose (79)]. Autumnal modifications in glucose metabolism cut across differences in photoperiodic species between those active nocturnally vs. diurnally, between obligate vs. facultative hibernators, and between nonhuman species vs. humans (80). One previously proposed reason for such autumnal metabolic change, that animals must maximize carcass fat to provide fuel for overwintering, works well for the Syrian hamster, a species that *gains* body mass in SD, but poorly for the Siberian hamster, one that *loses* body mass in SD (81). We have recently advanced an alternative suggestion, namely that the glucose requisitioned in autumn is shunted within specified seasonal CNS loci into the so-called aerobic glycolysis (AG) pathway so as to yield a number of end-products including but not limited to lactate and ATP (80). Intriguingly, the glycolytic pathway, much more active in astrocytes than in neurons (82), generally runs to completion within some 2–4 min (83, 84). Since glycolytic products including lactate and ATP can feed back powerfully within the glial syncytium to potentiate an initially weak signal (see below), AG may comprise the iterative generator of substances that not only label SDs as such but also gradually amplify the originally marginal SD signal.

### Aerobic Glycolysis as an Interval Timer

We are proposing that the relationship between the time taken for AG to run to completion inside astrocytes and the temporal extension, from 1 day to the next, of autumn scotophase is not fortuitous. *In other words, successful completion of AG serves in the initial phases of the autumnal response as a surrogate for the supra-2-min sidereal SD signal*. Thus AG in itself may be said to comprise a mechanism long intuited as necessary for seasonal physiology but rarely given a concrete housing either molecular or anatomic: an interval timer (85). Yet the timer as elaborated within our hypothesis differs in a crucial respect from the classic one: students of seasonality generally ascribe to the interval timer construct the capacity to distinguish an absolute duration of time, e.g., that which in the Syrian hamster labels a melatonin pulse as exceeding 11.5 h and thus as competent to trigger the SD response. Our interval timer, on the other hand, is a comparative one, measuring the *difference* between yesterday's duration of scotophase and today's and, when that difference regularly exceeds 2 min, inviting the organism to roll out or continue its SD involutional process. Thus it is not only AG as such that allows us to highlight the role of astrocytes in photoperiodism: it is also the relatively slow temporal scale, measured in minutes, on which astrocytes carry out certain functions vs. the millisecond scale preferred by neurons (83, 84) that renders the former cell type a central actor in seasonality. Before turning to the role(s) of AG in the seasonal network downstream from its interval timer function, we need to understand the molecular factors that contribute to the successful accomplishment of this cascade.

To drive glucose through the AG pathway, the astrocyte requires sequential or near-simultaneous stimulation of two different second-messenger pathways, one involving calcium signaling and the other cyclic AMP (cAMP). This has recently been demonstrated clearly for NE: through α1 receptors it generates a calcium signal that sets AG in motion; through β2 receptors it activates cAMP which serves to prolong and maintain AG (86). Dopamine is capable of the same complex yoking of calcium and cAMP signal cascades, stimulating the former in part through nonreceptor-based means (see below) and the latter primarily through the D1 receptor. The “synarchic” linkage of calcium and cAMP cascades, one that also figures in a number of nonphotoperiodic contexts, e.g., insulin granule exocytosis (87), will prove useful when we explore the anatomy of the seasonal response. Now both NE and DA are generously represented within the seasonal circuitry outlined above—especially within the aBNST node which likely harbors the greatest density of NE fibers within the mammalian brain (88). Yet while NE and DA are, we believe, the primary photoperiod-dependent activators of AG in the aBNST, they are not the only ones. This complex region serves as a convergence point for a number of peptides with the capacity for “synarchic” coordination of calcium and cAMP cascades. Of these, we mention only VIP which besides possessing that dual capacity (89, 90) projects to the aBNST from several loci (91–93) including the vlPAG. There, it colocalizes with a subset of DA A10dc neurons adjoining the cerebral aqueduct (94) and, significantly, aids in organizing REM sleep (95). To simplify matters, we largely ignore these peptidergic supporting actors, focusing instead on the aBNST-resident monoamines. We now turn to the aBNST itself, a region that, we believe, plays a central role in sculpting the mammal's response to autumnal SD including its capacity for bivalently modulating arousal.

## The Anterior Bed Nucleus of the Stria Terminalis (aBNST) as a Substrate for Glycolysis-Driven Seasonal State Changes

### Arousal-Related Switches Implicating the BNST

In recent years the aBNST has received escalating attention as a substrate for mood disorders (96). Further, it has decisively been implicated in at least two different forms of nonseasonal phenotypic switching, *each involving the dimension of arousal*. First, it is now well established as a locus for a species of neurobehavioral switching that appears an almost inevitable component of drug abuse. The strong DA A10dc projection into the anterolateral BNST [alBNST], especially the oval subnucleus (97, 98), clearly participates in drug-induced hyperarousal (99) many symptoms of which have obvious similarities to manic euphoria (see below). Yet induction of hyperarousal by drugs such as cocaine is regularly succeeded by a negatively valenced state of withdrawal, frequently amounting to frank depression (100, 101). While the circuitry underwriting the negatively valenced state remains less clear than that subserving the hyperarousal phase, a leading candidate is the medulla-based NE system that ramifies densely within the anteromedial BNST (amBNST) (102, 103). (For both NE and DA projections to the am- and alBNST respectively, see Figure 2) Similarly, the same NE→ amBNST circuitry provides a key component of the involutional syndrome that follows classically upon infection (“sickness behavior”) (104). Each of the amBNST-affiliated hypoarousal phenotypes is strongly reminiscent of the involutional phase of the SD response. (The classic hypersomnia of the typical SAD patient in winter is also a feature of “sickness behavior.”) We thus have preliminary warrant to align DA- and NE-driven aBNST events with the hyper- and hypoarousal phases respectively of the SD response. Since the autumnal organism begins with hyperarousal as a preamble to involution (see Introduction and Figure 1) and since DA is frequently identified as a sponsor of arousal, we begin with DA and the alBNST.

### Hyperarousal First: The Work of DA in the alBNST

Dopamine has long held pride of place as a molecular correlative of the manic phase of bipolar disorder (105). Pressured speech, hyperkinetic motor behavior, insomnia, euphoria, elevated libido and propensity for reckless behavior, all classic signs of the manic episode, have collectively been referred to increased activity of midbrain DA systems, generally (for motor function) those based in the substantia nigra and (for non-motor aspects) the A10 neurons housed largely in the ventral tegmental area (VTA) (105). Less clear has been the contribution of individual DA A10 subsets. One candidate worthy of consideration is the DA A10dc cell group, that which projects lavishly into the alBNST and especially into its oval subnucleus (97, 98). As noted above, dopaminergic activity within the oval subnucleus correlates closely with the hyperarousal driven by cocaine and related street drugs (99). And the A10dc neurons support the long-sought dopaminergic contribution to the daily waking phase (106). Particularly relevant to the autumnal hyperarousal both of classic photoperiodic animals and of the human being with SAD is the proximity of A10dc neurons to the cerebral aqueduct (94, 97, 98). For this location renders them susceptible to modulation by CSF-borne melatonin which stimulates cAMP but only when its pulse is prolonged by SD (107). Furthermore, DA A10dc cells insofar as overlapping with the dorsal raphe nucleus may in some species comprise a target of a direct retino-raphe projection (108, 109) or a more recently defined indirect retino-vlPAG visual circuit (110). Perhaps most relevant to our model is the promise of the A10dc→ alBNST system as an *incremental* amplifier of arousal.

Dopaminergic fibers in the alBNST (97, 98) and elsewhere in the brain display a predominantly nonjunctional form of release, i.e., their release sites tend not be in apposition to postsynaptic receptors (111, 112). Once liberated from A10dc varicosities, DA promotes calcium shifts in astrocytes through both receptor- and non-receptor-based means, driving the latter through a step involving ROS created through autooxidation of DA itself (113, 114). *The resulting calcium signals can directly trigger AG and, subsequently, extracellular release of gliotransmitters including lactate, ATP, and glutamate* (115). In the nucleus accumbens, closely connected to the BNST (116), ATP release, augmented by hyperglycemia and insulin, subsequently liberates *additional* DA by acting through a P2Y receptor on DA terminals (117), illustrating positive feedback at the signal cascade level (DA→ ATP→ DA). Dopamine also impinges directly on BNSTov neurons bearing D1 receptors; these include corticotropin-releasing hormone (CRH)/GABA neurons which augment arousal (98). Furthermore, its likely modulation by both luminance and melatonin (see above) combined with its projection upon the LHA (106) renders the DA A10dc neuronal group an attractive candidate for the luminance-inflected vector that summates in autumn inside the LHA with the REM sleep-inflected vector, thus activating the LHA “incremental augmenter” (see How Would a REM-Based Luminance Search Device Work With the Supra-2-Min Constraint?). *Thus both directly and through the intermediary of astrocytes, DA release within the alBNST and other seasonal nodes could account for slowly growing organismal arousal through the entire pre-switch phase*^2^.

Another pro-arousal mechanism possibly allied to DA may involve the daily glucocorticoid (GC) pulse profile. Recall that sympathetic hyperactivity involving increased RSNA activity surfaces by 14 days of SD exposure and continues throughout autumn and winter (see The Supra-2-Min Problem). A cognate species of sympathetic activity, one mediated by the thoracic splanchnic nerve, impinges on adrenal and more specifically HPA activity (see Figure 3). This likely accounts for the phase-advanced and/or augmented GC pulse frequently displayed in autumn by photoperiodic animals (118–122) as well as by the bipolar patient (see below). [Interestingly, healthy adult humans display a strikingly phase-advanced salivary cortisol profile, although no alteration in amplitude, in winter vs. summer (123)]. In augmentation both of HPA activity and of autonomic (sympathetic) drive, the aBNST clearly plays a part although linkages between these two arousal-related effects and precise subnuclear contributions remain elusive (124–126). Without developing the case at length, we propose that ultimate responsibility for augmented SD sympathetic activity, whether that of interest to the Bartness group (see IIB) or that which phase-advances the GC pulse, rests with the DA A10dc projection into such established central autonomic nodes as the LHA and the alBNST.

As for bipolar patients, studies of their seasonal GC profiles have at times failed to distinguish *early SD* (i.e., autumnal) from *late SD* (i.e., winter) events. For example, one frequently cited paper on GC pulse dynamics in seasonally depressed SAD patients found a radically *blunted* GC curve (127); given that all serum samples were drawn no earlier than November, this testifies clearly to the later (i.e., winter), not to the earlier (i.e., autumnal), development. We suspect that the earlier, peri-equinoctial, situation involving the hypomanic SAD patient would resemble the previously noted GC profile in mania at large, i.e., elevated and/or phase-advanced (128, 129). Of special note is the significantly augmented GC pulse in patients with “dysphoric” mania (130, 131), an endophenotype for which the early SD aggressiveness of smaller photoperiodic mammals (see Introduction) may serve as a model. In any event the seasonally modified GC pulse bears crucially in two ways on DA dynamics as we approach the switch itself.

First, the degree of phase-advance of the GC pulse in the SD organism, whether classically photoperiodic or bipolar, is on the order of 30–60 min (118–122, 128, 129). *Such a phase-advance causes the GC acrophase to migrate across the border separating waking from sleep*. Thus instead of falling some 15–20 min into the waking phase, the pulse acrophase in SD is brought squarely into the center of the concluding epoch of sleep–i.e., the last, longest, and most vigorous REM sleep episode (see Figure 3). Of note, GCs are potent magnifiers of astrocytic calcium waves (132). *This attraction of the GC pulse acrophase into late sleep, which we see as a key precursor to the switch process, amplifies calcium wave activity already instigated in aBNST astrocytes by DA*. At least at first, such signaling would promote yet further release of DA.

The second effect of the modified GC pulse in SDs with implications for DA release stems rather from its augmented amplitude than from its phase-advance. As Gasser and Lowry have shown in detail, GCs powerfully inhibit the organic cation transporter (OCT), a high-capacity, low-affinity transporter mediating uptake of DA as well as serotonin (5HT), NE, and histamine (133). Interestingly, the distribution of the OCT3 overlaps significantly with the seasonal network, with strong representation in the DMN, the PeF, and the CVOs (134, 135). Such inhibition of the major high-capacity transporter of DA by GCs means that DA concentration (as well as that of other relevant monoamines) will remain significantly augmented within seasonally relevant nodes roughly during the acrophase of the GC pulse, i.e., during the last and longest REM sleep episode. Analogously, mice with reduced activity or knockout of the low-capacity, high-affinity DA transporter (DAT) display hypersensitivity to change of photoperiod, thus furnishing one of a small number of persuasive animal models for bipolar disorder (105, 136–138). One could pursue this promising and relevant model by developing a prototype that integrates both transporter species, the DAT and the OCT3.

Glucocorticoids, then, previously incriminated in the switch process (139), may be said to shift the nature of subcortical processing in SD both by augmenting astrocytic calcium signaling and by elevating monoamine levels^3^ within the glial syncytium. Although by promoting DA release this shift may initially support the hyperarousal phenotype [a well-known effect of exogenous GCs (140)], it may also serve as forerunner to the involutional state.

### Into and Through the Switch, a New Actor: The NOX Enzyme Family

We have already mentioned, as preliminary warrant for considering a link between seasonal involution and the NE-afferented amBNST, two other involutional phenomena connected with the amBNST, namely the quasi-depressive phase which succeeds drug-induced arousal and the “sickness behavior” precipitated by infection (see The Supra-2-Minute Problem). Our task here is to explore the possibility of a BNST-based mechanism by means of which the seasonal form of involution comes about. In perhaps simplistic terms, how does the action of DA in the alBNST get communicated to that of NE in the amBNST? And how does its behavioral polarity get reversed in the process?

It is at this point that NOX enzymes and their ROS product come into view. That ROS have a role in seasonal switching is hardly novel. Indeed NOX enzymes, which have a long evolutionary history (141), participate in seasonal modulation of life forms including many plants (142) and invertebrates (143) that evolved well before mammals. And in amphibians NOX-derived ROS contribute to different kinds of phenotypic switching at large (i.e., not specific to photoperiod) such as that involving Xenopus tadpole tail regeneration (144) and amphibian metamorphosis (145). Until recently, their position in mammalian photoperiodism has been less clear. However a physiological role of NOX-derived ROS has emerged over the past decade with regard to mammalian neurogenesis—and here we approach links to seasonality proper.

The phenomenon of neurogenesis—the differentiation of a stem cell into a neuron—may represent the most radical form of CNS plasticity. Research on DA neurogenesis suggests that the key DA progenitor/stem cells tend to arise in the dorsocaudal midbrain, i.e., roughly the region that eventually will house the DA A10dc cells. They then migrate from that location to populate the sectors that with maturation of these progenitors will become the substantia nigra pars compacta and the VTA (146–149). Significantly, one powerful influence upon neurogenesis involves photoperiod (150). Now DA neurogenesis appears to have a tropism for *SD* that cuts across distinctions based on breeding season (151). Why should neurogenesis be conjoined temporally with the involutional phase? At least a proximal answer may involve the roles which amBNST NE plays in both of those processes.

As recently emphasized (152), the noradrenergic β2 adrenoreceptor (β2AR) affords a functional link between NE and NOX activity. While clinical applications have heretofore been limited, such a link sets the stage for understanding how NE may contribute to the prosurvival phase of the SD response. Working through ROS, NE is well poised to drive activation of one of the major neurotrophic substances, brain-derived neurotrophic factor (BDNF), which in turn safeguards the viability of DA neurons (in this case, through winter) (153). Now although NE itself can directly activate transcription of BDNF (154), its role in the seasonal narrative would be more subtle: it would begin by transactivating, through ROS, the TrkB receptor, one of the two main receptors for BDNF. Such transactivation — the activation of a receptor tyrosine kinase independently of the presence of its traditional ligand (155)—is well suited to the incremental nature of the SD response. For transactivation of a RTK yields a more modest response than ligation by the traditional agent (in this case, BDNF) (155); this accords with the initially imperceptible beginning of the SD response. Direct signaling by BDNF protein, known to be transported in anterograde fashion from the lateral parabrachial nucleus (LPBN) to the alBNST (156), would follow at a later stage. And if in early life the BDNF carried by NE fibers to the ventral midbrain DA progenitor cells promotes DA neurogenesis (157), in maturity the same ligand could account for the neuroplastic (if not frankly neurogenetic) effects, i.e., those involving a neurotransmitter switch, now well documented in photoperiodism (158–160). Through its potent bidirectional connections with both hypothalamic (161) and brainstem (162) arousal sites, the aBNST thus offers possibilities not only for rapid-response modulation of arousal but also for participating in slow transport of neurotrophic ligands such as BDNF^4^.

**Figure 4:**
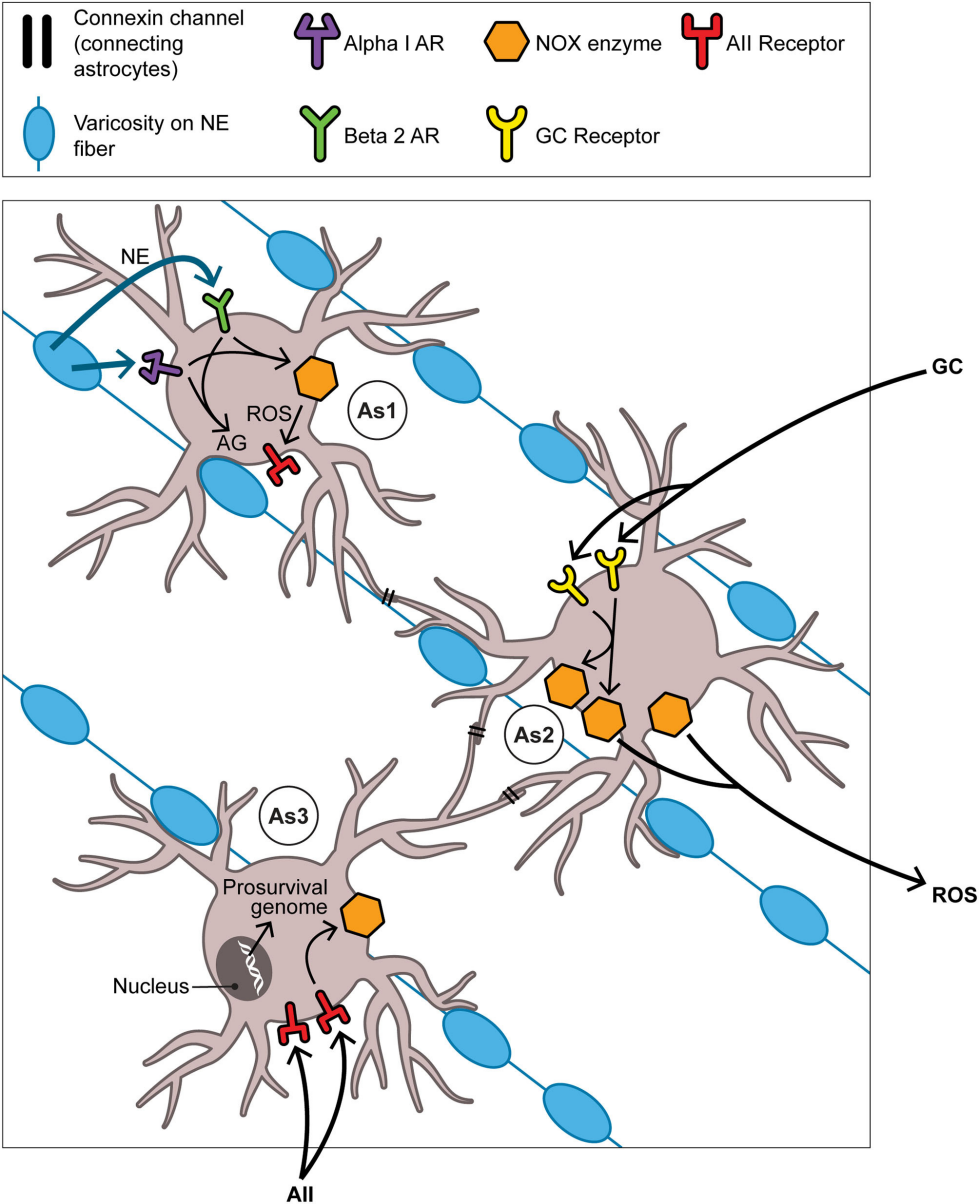
The aBNST-, Astrocyte-, and NOX-based cycle: focus on the full autumnal switch. As1, As2, and As3 represent astrocytes in the amBNST; their processes envelop many of the NE varicosities which obtain in abundance here. Each astrocyte should be understood to dominate events primarily, though not exclusively, in one of three temporal intervals: As1 in the pre-switch or hyperarousal phase, As2 in the intra-switch phase, As3 in the post-switch or involutional phase. That each one of the NOX enzyme activators (NE in time 1, GCs in time 2, AII in time 3) contributes differentially to the generation of ROS in its given interval remains, of course, a heuristic construct. This is not incompatible with the notion that the magnitude of the ROS signal gradually increases as the phases succeed one another. As to the ultimate target of the ROS signaling depicted here, the text argues that this involves the neurotrophic or prosurvival program. However this does not exclude subsidiary engagements, some but hardly all of which are discussed in the text, with other aspects of the hypothesis. For example, the NOX system likely meshes with the process of AG that inaugurates the SD response. Briefly, the pentose phosphate pathway (PPP), branching off from the glycolytic pathway, produces NADPH, the reducing equivalent required by NOX. Thus although the PPP is frequently cast as antioxidative in nature through its sustenance of the robust antioxidant glutathione peroxidase, it nonetheless manages in certain contexts to provide NADPH in controlled support of the oxidative NOX system. Abbreviations not previously mentioned in figure captions: AII, angiotensin II; AG, aerobic glycolysis; AR, adrenoreceptor (alpha1 or beta2); ROS, reactive oxygen species.

The neuroplastic/neurogenetic potential of the aBNST is not likely to be limited to that driven by NE. We can only glance at one of several other promising conduits for neurogenesis in the BNST and connecting regions, namely that involving the fibroblast growth factor (FGF) family of neurotrophins. Reactive oxygen species themselves are capable in the retina and elsewhere of directly transactivating the FGF receptor and thus underwriting a prosurvival program (163). Indeed FGF neurotrophins serve as regulators of stem cell pluripotency (164) and have a growing role in seasonality given that FGF21 in particular figures in SD responses including torpor (165) and hibernation (166). The number of possible activators of NOX enzymes and of downstream permutations renders it hazardous to arrive at a possible sequential order for events pertaining to NOX-derived ROS generation and succeeding signaling events over the many weeks of the mammalian SD response. Nevertheless in Figure 4 we venture to propose such a temporal order with focus on NE, GCs, and angiotensin II (AII) as activators of aBNST NOX and of the seasonal switch. Here we depict ROS as products of astrocyte-based NOX enzymes; once created, these ROS may then be active both intracellularly and, after transport through aquaporin channels into ISF (167), extracellularly as well.

### REM Sleep-Related Acetylcholine as One Guardrail Delimiting Seasonal Events in the BNST

As we have suggested at intervals, the generation of ROS within an animal brings with it significant risks: while carrying out their physiological assignment, they may cross over into pathological territory. The seasonal animal has two ways of addressing this risk. It can (a) generate a robust antioxidant response or (b) actively limit the production of ROS. Postponing the topic of (a) for now, we address (b) here—the confining of ROS generation to as modest a spatial and temporal footprint as possible. This implies fencing seasonal ROS production spatially within the defined photoperiodic module and temporally within one particular tranche of circadian activity, namely REM sleep. We now tie up a loose end by proposing that the acetylcholine associated with REM sleep has an important “guardrail” assignment concerning this spatiotemporal strategy. In doing so we connect with an older theory concerning bipolar biology.

Cholinergic fibers originating largely within two brainstem cell groups, the pedunculopontine tegmental nucleus (PPN) and the laterodorsal tegmental nucleus (LDT) (168) ramify densely through the amBNST but fail to enter the oval subnucleus of the alBNST (169). Although no longer considered an exclusive driver of REM sleep, cholinergic activity associated with these 2 pontine nuclei remains an important actor within that sleep phase. Increased cholinergic activity in REM sleep, often greater than in waking (170), is well placed to facilitate NE release within this division of the aBNST through a nicotinic presynaptic action on the abundant NE varicosities here. If one grants the assumption that such presynaptic stimulation of NE generally requires cholinergic levels consistent with REM sleep, then we may conclude that the NE ultimately responsible for generating neurotrophic factors such as BDNF in the amBNST is released within but not outside of REM sleep. Note that such spatiotemporally restricted release of NE within the amBNST, no doubt the result of vigorous calcium signaling here, may transpire independently of action potentials triggered within the noradrenergic neurons projecting to the amBNST^5^. Such “chemical” stimulation of NE release independently of action potentials has been documented in the aBNST (171). Our model thus respects the finding that the ventral medullary NE neurons are as electrically silent in REM sleep as those in the LC (172). Further, the PPN/LDT→ amBNST cholinergic projection (omitted from Figure 2) likely takes part in a recursive anatomy including an incremental augmenter running parallel to the one proposed earlier (see IIC). For neurons in both the al- and the amBNST return a projection directly to the PPN (173) and LDT (174). This pathway offers the seasonal module another means of iteratively amplifying some component of REM sleep such as REM density^6^.

Such a function for ACh in tightening the intimate relationship between the SD photoperiodic response and REM sleep (which obtains according to our model in seasonal mammals at large) bears a qualified resemblance to that which it has in the bipolar model proposed some years ago by D. Janowsky (175). [The latter model applies only to human beings with BD although a similar conception finds support in recent work pertaining to seasonal animals (176)]. According to the Janowsky model, cholinergic activity comprises a prime mover of the depressive phase of BD. At first glance at least, our model does something similar in that cholinergic activity provides a proximate cause of the NE release that in turn drives neurotrophic factors affiliated with depression. Yet in our model Ach ultimately does something more ambitious. For insofar as clearly *upstream* from the NE/NOX/neurotrophic axis, ACh in effect functions as an integral part of the switch mechanism. That is, it contributes to a process of slow maturation involving NE release which *chronologically precedes and partly causes* the involutional phase—originating, in ways that remain to be fully determined, within the hyperarousal stage that serves as preamble to involution (Recall that NE has a hand in the AG which figures in both phases). Our model thus addresses a weakness in the Janowsky model highlighted in a recent review, namely that it does not in and of itself account for the switch process (175). Far from claiming too much for cholinergic activity, the Janowsky model did not claim quite enough.

To summarize the argument of Section The Anterior Bed Nucleus of the Stria Terminalis (aBNST) as A Substrate for Glycolysis-Driven Seasonal State Changes to this point, *the general seasonal mammalian switch centers on a BNST-grounded, NOX-assisted, and REM sleep-delimited mechanism. This mechanism closely resembles or may be identical with that which governs the switch, as described by students of neurogenesis, from a proliferative to a neuroplastic/neurogenetic program* (146–149). Although human beings with BDII/SAD hardly inherited the entire photoperiodic constellation, they did retain, we have argued, the core seasonal switch process. Thus through their complex symptomatology we may discern a significant remnant of the bifold modification of arousal evident in nonhuman seasonal mammals. It may be proposed that the human aBNST, like that in nonhuman organisms, stages the turn from manic hyperarousal to depressive—and prosurvival— involution.

### Revisiting Once More– and Transcending– the Supra-2-Min Condition

By invoking a robust transcriptional component, especially in regard to the generation of neurotrophins BDNF and FGF, as contributing to the seasonal switch from hyperarousal to involution, we may seem to have violated our own earlier stipulation involving a supra-2-min constraint on biochemical cascades pertinent to SD. For transcriptional events, of course, must be measured in hours and not minutes. Yet the contradiction is superficial. Photoperiodism operates simultaneously within two different time frames, the first involving rapid and nongenomic events and the second transcriptional events. We have abundant evidence that a rapid and nongenomic response can not only coexist but indeed mesh seamlessly with a later genomic one. Of note, some of the most striking marriages between nongenomic and transcriptional responses involve GCs (177). Indeed a full third of cases of GC-driven transcription pass through a rapid nongenomic stage (178). And DA signaling through D1-D2 heteromers leads to a dramatic cytosolic calcium signal followed within hours by transcription of the BNDF gene (179). Further, it should not be surprising that the late SD phenotype differs strikingly from the earlier one.

For the seasonal organism, as we have emphasized, must limit some of the dangers intrinsic to the early phases of the SD response. We have previously reviewed several ways by means of which the photoperiodic animal minimizes the production footprint of ROS. Here we discuss its investment in a complementary strategy, namely the development of a robust antioxidant response. Not so paradoxically, many of the agents and processes mentioned in conjunction with the current ROS hypothesis may themselves function as antioxidants. This includes NE itself (180) and CRH (181). Above all perhaps, a family of antioxidant responses building on several sequential transcriptional steps must eventually come into play. The Nrf2 transcription factor, itself activated by BDNF, proves crucial in developing such responses (182). We cannot discuss here how events downstream from Nrf2 include not only the development of an antioxidant system narrowly defined but also promotion of behavioral involution. Such a powerful system evolved not to extinguish ROS but rather to increase their specificity as neurotrophic signaling agents while at the same time guarding vigilantly against off-target effects. The notion of an oxidative trigger as a means of stimulating a complex response (including but not limited to an antioxidant program) is consistent with the general concept of hormesis. According to this concept, a relatively low and transient dose of an otherwise noxious substance calls forth a response the beneficial effects of which outweigh the risks associated with the initial trigger (183, 184). Yet whether such a beneficial arrangement would survive nontrivial modification of the luminance environment in which it evolved is another question.

### Adjudicating Two Views on ROS and Related Inflammatory Mediators in Bipolar Disorder

Certain off-target effects of ROS signaling may have surged into prominence surprisingly recently. Mammalian photoperiodism was likely well advanced by some 50 million years ago amongst the small species proliferating in the Cenozoic era (185). Given the non-negligible remnant of seasonal modulation in modern man, it is conceivable that a somewhat more extensive version of photoperiodism obtained in an earlier hominid species. Indeed a plausible case has been made that Neanderthals exercised a general photoperiodic capacity (14). Yet even if that preliminary brief should prove true, this only postpones the question why part of the seasonal machinery was diverted into pathology. Conceivably the very sensitivity to luminance signals which grounds traditional photoperiodism would serve as an invitation to pathology were such signals delivered excessively or on an aberrant schedule. Particularly worrisome would be a luminance pattern that degrades the profound point of rarity between light and darkness that obtained until well into the Anthropocene. Indeed we have evidence that contamination of deep scotophase by man-made light can prove neurotoxic (186–188). With anthropogenic “light pollution,” then, ROS of the destructive kind may have entered into competition with the physiological species.

This brings us to the relationship between the hypothesis presented here and a lively current topic, namely that of “neuroprogression” in BD. As applied to BD, neuroprogression means the cumulative damage, over multiple episodes, wrought within the bipolar brain by inflammatory processes including oxidative ones. As such, this topic recapitulates and focuses the theme of ROS as damage agent, integrating this with material on related mediators of chronic inflammation. As noted at the outset, there is no reason to doubt the robustness of the evidence for damage to the brain of the patient with BD–especially the one who has experienced multiple episodes. Yet it seems to us that students of bipolar neuroprogression generally fail to consider the possibility that the inflammatory cascade driving the destruction of brain tissue represents an adulterated form of a normative —in our view, a seasonal—survival strategy. If, however threatened presently, that strategy has held sway for 99+% of the time that mammals have existed on earth, it becomes imperative to understand the nature of the connectivity between the original pro-survival template and its apparently recent divergence into pathology. We can begin by noting that certain factors apart from ROS are common to both of these developments.

One of these involves insulin resistance. Recent exciting work suggests that insulin resistance is at the core of the insidious decline comprising bipolar neuroprogression (189, 190). Yet insulin resistance also figures in the SD response. In developing our hypothesis, we have mentioned only some facets of the role of insulin and related agents. Highlighted, for example, was the hyperglycemia of the autumnal mammal, something that cuts across many different variants of photoperiodism (see A Seasonal Role for AG). Left unmentioned was a role for concurrent insulin elevation, attested in ground squirrels by a 4-fold increase in September-October (i.e., in prehibernation) (191). This has been viewed as linked with peripheral insulin resistance and facilitation of prehibernation weight gain (192). Thus mammalian hibernation has been put forward as a reversible model for the insulin resistance of type 2 diabetes (192, 193). While almost surely comprising a common part of the SD response, prehibernation weight gain does not apply to all seasonal mammals (see A Seasonal Role for AG); thus we suspect another role, applicable to mammals in general, for autumnal insulin spiking. One such role, central rather than peripheral and more directly opposite to our model, would involve a concerted action, along with insulin growth factor-1 (IGF-1), upon glucose uptake by astrocytes (194, 195). With entry into the brain and indeed astrocytes, we gain admission to a feature that distinguishes constructive from pathological ROS, glucose, insulin, and insulin resistance—the possibility of *cycling*. For cycling on a seasonal plane is precisely the solution that photoperiodic mammals began to exploit in the late Mesozoic so as to keep inflammation to an absolute hormetic minimum—so as *not* to enter upon the slippery pathway to cumulative damage.

In summary, our theory makes three predictions regarding the nature of the relationship between seasonal vs. pathological insulin resistance (and related inflammatory mediators including ROS). First, it suggests that a point of rarity, evolutionary and otherwise, may be discerned between these two processes. Despite significant overlap at the molecular level, an overlap that permits us to understand how and why the two are connected at all, significant damage obtains *regularly* in the older bipolar patient but *rarely* in the seasonal nonhuman mammal. Second, no differentially acting factor explaining the difference between these two outcomes need be invoked *other than* that involving two variants of luminance signaling—scrupulously photoperiodic vs. degraded or corrupted^7^. Third, the seasonal form of insulin resistance sorts with the cyclical generation of ROS by NOX enzymes whereas the pathological version sorts with ROS release in adventitious and “noisy” fashion from damaged mitochondria. In Box 2 we propose several experimental approaches whereby these predictions may be tested.

Box 2 **Suggestions for exploring the Interface between Photoperiodic (NOX-Generated) vs. Destructive (Mitochondrial) ROS.**

1. In a classic seasonal animal such as the Syrian hamster, one might examine the roles of NOX assembly and ROS production while the animal is exposed to SD (ideally naturally changing, otherwise fixed). If ROS in effect are produced within the aBNST under this photoperiod as our hypothesis predicts, one could then determine whether they originate with NOX, whether NOX is driven directly or indirectly by calcium signals within the BNST, and whether its efficacy as a generator of ROS is augmented as SD progresses. This experiment could then be repeated with a cohort of hamsters exposed to a “corrupted” SD photoperiod, e.g., one in which traditional scotophase, devoid of light, is replaced by dim, intermittent, or otherwise aberrant luminance. Our hypothesis would predict that animals under corrupted SD would display the kind of oxidative damage described in human beings with BD II/SAD whereas those under a pristine SD photoperiod would not.

2. Recent evidence for a significant phase-advance of the cortisol pulse in *healthy* humans in winter ves summer (see Hyperarousal First: The Work of DA in the alBNST; the samples were drawn in February and June, not a classic SD vs LD design) may serve as an excellent introduction to a physiological form of seasonality in modern human beings. Utilizing known analogues for such phase-advance in small seasonal animals (see Hyperarousal First: The Work of DA in the alBNST), one could attempt to define (a) the precise mechanism responsible for this isolated neuroendocrine shift, one that does *not* disturb the circadian activity rhythm; (b) the extent to which the phase-advance of the GC acrophase succeeds in positioning it onto the last REM episode; (c) the extent to which this phase-shift is a necessary precondition of later SD developments. Repeating aspects of this study with animals in corrupted SD (see previous entry) would help to answer the question whether corrupted luminance comprises a necessary and sufficient cause of oxidative damage within the brain.

3. Recent imaging studies of BD patients have demonstrated an increased lactate signal in the anterior cingulum (among many other papers, see Wang et al. [9]). Such a signal has been generally interpreted as supporting the mitochondrial dysfunction hypothesis of BD pathophysiology. Insofar as the studies were harvested in the waking phase and concern a cortical region, one moreover afferented by the LC noradrenergic system and not the ventral medullary one, they cannot be directly compared with our model. Yet careful study of the anterior cingulum in a photoperiodic context may cause one to question the mitochondrial interpretation. It is intriguing, for example, that the anterior cingulum (or, in the rodent analog, the infralimbic cortex) is positioned directly upstream from at least two major seasonal nodes, the LHA and the aBNST. Conceivably the cingulate cortex, insofar as a specialized area of neocortex and not a more primitive structure, evolved in part to *mitigate* seasonal modulation by the downstream photoperiodic module. Our hypothesis predicts that some relationship, even in the form of mitigation of the seasonal network by the anterior cingulum upstream from it, will be found to obtain; accordingly, the lactate signal in the cingulum results not from mitochondrial dysfunction per se but rather from its complex blend with photoperiodic function.

4. A recent provocative finding, involving the first study of blood-brain barrier (BBB) function in living human beings, points to regular breaching of the BBB in the “late” bipolar patient. (See Kamintsky et al. [190]). Such dysfunction of the BBB, while strongly consistent with the neuroprogressive paradigm, may point to a link with our hypothesis. For insofar as the BBB comprises a significant component of the CVOs, its breaching in the modern BD patient may represent degradation, under oxidative damage, of that important component of the seasonal apparatus. Our hypothesis would predict that such breaching occurs *rarely* in the photoperiodic animal proper and *frequently* in the same animal exposed to a corrupted luminance schedule. This could be explored utilizing a classic photoperiodic mammal such as the Syrian hamster. Although much less frequently utilized, the rhesus macaque monkey is also photoperiodic and could therefore help to determine the parameters of such BBB function and dysfunction in a nonhuman primate.

5. Another exciting field in bipolar research with possible relevance to our hypothesis involves the theme of regulated exocytotic release of signaling molecules contained within membrane-bound vesicles that are exported from both neurons and astrocytes. We now have evidence that astrocytes and neurons obtained from BD patients display expertise in this form of transmitter release. (For a quick orientation to this field, one may search PubMed for work by K. S. O'Shea for years 2013-2022.) Such exocytotic release is felt to have both physiological and pathological affiliation. We suspect that one of its physiological affiliates involves seasonal (photoperiodic) modulation of function. Factors pointing in this direction include: (a) the relatively slow nature (requiring minutes) of exocytosis from astrocytes (see Verkhratsky et al. [83] and Zorec et al. [84]), a fact that renders this process an excellent candidate for distributing the products of AG; (b) the frequent use of extracellular vesicles for secretion of growth factors (see Zorec et al. ibid.) consonant with the involutional phase of the SD response. Our hypothesis predicts that vesicular exocytosis of substances including FGF transpires *regularly* within the REM sleep phase of the autumnal mammal and contributes to maturation of the SD response but *chaotically and “noisily”* in patients with BD where it is disconnected from that particular temporal niche and feeds progressive inflammation.

## Conclusion

### How Does the Current Photoperiodic/Bipolar Switch Hypothesis Differ From Previous Ones?

Our approach to the problem of the photoperiodic/bipolar switch process separates from previous ones in three ways. First, it establishes a more detailed fabric of continuity between the classic photoperiodic animal and the seasonal bipolar patient than has obtained thus far. The common denominator linking these two mammals resides less in the particular features of the organism at a given season (hypersomnia can only metaphorically be termed hibernation) than in the hinges or transition points separating the seasons from each other. Thus the articulation between autumn and winter involves roughly the same switch-related signal cascades in the Syrian hamster and the human being with BDII/SAD. Highlighting the role within these cascades of NOX-derived ROS marks a divergence from the current emphasis in BD research upon ROS as agents of pathology. As noted above, the two aspects of ROS may well coexist in the modern patient: the first or constructive aspect, dominant throughout mammalian evolution until the very latest phase of the Anthropocene, may by means of its exquisite sensitivity to ROS have prepared for the advent of the deleterious kind. It will take work to disentangle, within that patient, the relative contributions of the two.

Second, we have proposed partly novel conceptualizations of the roles both of REM sleep and of astrocytes in mammalian photoperiodism at large. Rather than responding passively to sidereal change, the mammal in our formulation searches actively and continuously for evidence of such change during the crepuscular interval which necessarily coincides with its last and longest REM sleep episode. Exploiting the temporal equivalence between the daily quotient of scotophase extension in autumn and the running to completion of AG, the seasonal organism deploys a largely astrocyte-based interval timer which renders the generation of glycolytic products contingent upon a particular photoperiod. On the diurnal time scale, export of products of AG is viewed as taking place during the same REM sleep episode proposed for the animal's interrogation of crepuscular luminance. To be sure, the resulting conception of AG as transpiring in sleep within selected astrocytic microdomains puts us at odds with one aspect of the glymphatic system concept as currently elaborated. For the latter positions AG strictly as a waking-phase and cortical cascade driven by LC-grounded (not medullary) NE (196). However in many other respects we remain indebted to the glymphatic hypothesis. By incorporating a second noradrenergic focus involving the medullary system, the glymphatic metaconcept can be largely reconciled with the present model.

The third fundamental difference involves a significant revision of the anatomic substrate of mammalian seasonality. The aBNST is brought forward as an integral component of the seasonal network, one which communicates closely with previously documented nodes both by hard-wired pathways and through volume transmission taking place primarily in sleep. Aligned in multiple ways, both moieties of the aBNST (medial and lateral) together carry out a behavioral switch process permitting the organism to survive winter.

### What Does the Model Omit?

Our hypothesis, the main features of which are listed in Box 3, can be taxed with any number of omissions. Hardly given their due, or even mentioned at all, are many likely actors in the photoperiodic process (some of which, intriguingly, do figure in recent work on the BNST). This includes pituitary adenylate cyclase activating polypeptide (PACAP), Klotho, nitric oxide, leptin, RFamide-related peptides, β-arrestins, hypoxia inducible factor-1α (HIF-1α), dopamine and cAMP-related phosphoprotein-32 (DARPP-32), GABA interneurons and clock genes within the extended amygdala, tissue plasminogen activator (tPA), the Wnt/β-catenin and insulin/insulin growth factor/PI3K signal cascades, and no doubt others. But if the model can serve heuristically to guide further research into the means whereby the photoperiodic mammal develops its remarkable response to seasonal change as well as into the manner in which human beings both inherited and mitigated this response, then the effort will have been worthwhile.

Box 3 **Main Features of the current photoperiodic hypothesis.**

1. In photoperiodic mammals, one function of REM sleep involves active search, within the last and longest REM sleep episode, for evidence of changing luminance signals, i.e., an incursion into that episode of a scotophase longer than that which obtained on the previous day. (See “Crepuscule Change Detector” in Box 1.) The animal's encountering such evidence when of sufficient magnitude activates a gradual process of involution that will take weeks to mature.

2. As one consequence of the animal's SD response, DA neurons in the A10dc subset release DA into the neuropil of the alBNST, their primary target. This leads early on to functional recruitment of the alBNST into the seasonal network. At the same time these DA neurons increase calcium signaling in the alBNST and thereby activate AG in astrocytes.

3. Subserved primarily by the dopaminergic projection into the alBNST, the hyperarousal phase of the SD response (analogous to the mania or hypomania of bipolar patients) ensues. This involves daily augmentation of the amount of DA liberated within the alBNST. During this time spatiotemporal boundaries to the activational process are defended insofar as AG is activated and its products distributed only in REM sleep and only in the seasonal module.

4. At some point, certain subtle changes begin to prepare a phase reversal in the behavioral polarity of the seasonal animal. Schematically, this may involve a shift in the prime staging area of the SD response from the al- to the amBNST and from DA to NE as presiding monoamine. On the neuroendocrine front, phase-advance of the GC pulse leads to its full attraction into REM sleep and thus to maximal signaling of GC-sensitive elements, particularly astrocytes, within the amBNST. Maximal presynaptic release of NE here through “chemical” depolarization liberates NE, working through the β2 adrenergic receptor, which in turn drives NOX enzyme assembly and generation of ROS. Working as part of a well-defined signal cascade, these ROS begin to transcribe a prosurvival genome set including neurotrophic factors such as BDNF and FGF. The ensuing genomic shift may be described as one from a proliferative to a neuroplastic/neurogenetic gene program. Dissemination of neurotrophic factors through the glial syncytium and through anterograde neuronal transport conduces both to the survival of DA neurons and to their reversible functional decline consistent with the involutional phase of the SD response.

5. Hominid species gradually had to curtail full adoption of the seasonal program. Nevertheless a modified photoperiodism may have obtained among early hominids (see Revisiting Once More—and Transcending—the Supra-2-Minute Condition). In the modern human being with BDII/SAD, seasonal modulation represents an atavistic remnant of photoperiodism.

6. In a remarkably recent development, modern humans introduced “light pollution” into their environment. This led to degradation of traditional seasonal signaling. The modern human being with BDII/SAD represents a complex amalgam of the old, physiologically serviceable, photoperiodism in which ROS figure as an actor in a signaling cascade with a new overlay resulting from ROS in its guise as a damage agent.

## Data Availability Statement

The original contributions presented in this study are included within the article, further inquiries can be directed to the corresponding author/s.

## Author Contributions

The author confirms being the sole contributor of this work and has approved it for publication.

## Conflict of Interest

The author declares that the research was conducted in the absence of any commercial or financial relationships that could be construed as a potential conflict of interest.

## Publisher's Note

All claims expressed in this article are solely those of the authors and do not necessarily represent those of their affiliated organizations, or those of the publisher, the editors and the reviewers. Any product that may be evaluated in this article, or claim that may be made by its manufacturer, is not guaranteed or endorsed by the publisher.

## Footnotes

^1^For technical terms such as photophase and scotophase, see Box 1, Glossary of Selected Terms.

^2^We cannot discuss within the confines of this paper an apparent conflation here of REM sleep arousal with waking-phase arousal. Doing so would involve adducing the increasingly well-documented stimulatory effect of midbrain DA neurons upon REM sleep. Dopamine, we believe, sequentially or simultaneously activates both forms of arousal.

^3^The role of the monoamine serotonin in seasonality, a topic which has received much attention, cannot be addressed here.

^4^Note that the role posited here for BDNF within the involutional phase appears to contradict that which this neurotrophic factor frequently has in mediating antidepressant treatments (not limited to medication). The contradiction disappears once we take into consideration the circuitry involved. Nearly all the studies of the antidepressant role of BDNF involve the hippocampus; conversely, the involutional (simplistically, pro-depressant) activity in our model involves the BNST portion of the central extended amygdala. These two circuitries have different and sometimes opposed responsibilities. This stems in part from their radically differing NE innervation: the LC provides the entirety of the hippocampal NE; the medullary NE system supplies nearly all of this monoamine to the BNST.

^5^Since the GC pulse contributes materially to calcium signaling, its proposed relocation under SD into REM sleep (see IVB) suggests that it takes part along with other actors in restricting vigorous NE release to this sleep phase.

^6^For convenience, we overlook certain complexities in the manner in which REM sleep-affiliated Ach stimulates NE release. This likely involves an astrocytic intermediary and subsequent discharge of glutamate onto NMDA receptors which are plentiful on NE terminals in the amBNST. These complexities in no way hinder the proposed linkage of NE discharge into the amBNST with REM sleep. Finally, the rather striking step-off in Ach fiber density going from the am- to the alBNST (i.e., from generous to nearly absent) is consistent with the animal's maintaining a measure of independence between two different vectors, one associated with REM sleep and the other with a luminance signal, segregating with the amBNST and alBNST respectively (for these vectors, see IVB). True, these signal trajectories were represented as summating under specified conditions within the LHA in order to inaugurate the SD response. But that is a contingent matter, emerging only when a SD luminance signal coalesces with a REM sleep signal. It would be counterproductive to link the REM sleep vector *constitutively* to the luminance vector. And so cholinergic fibers avoid the oval subnucleus, closely connected, in ways noted above, with processing of luminance signals.

^7^Or, if any other cause should be discovered, it will involve a substance or influence with as recent a history as anthropogenic light pollution. One such cause may involve the ready dietary availability of simple sugars. Casual and steady consumption of exogenous sugars would likely overwhelm the photoperiodic mechanism, one based primarily on a jealously guarded astrocytic storehouse of the glucose polymer glycogen.

## References

[1] Blumberg HP. Euthymia, depression, and mania: what do we know about the switch? Biol Psychiatry. (2012) 71:570–1. 10.1016/j.biopsych.2012.02.003 22424111 PMC3874046

[2] Young JW Dulcis D. Investigating the mechanism(s) underlying switching between states in bipolar disorder. Eur J Pharmacol. (2015) 759:151–62. 10.1016/j.ejphar.2015.03.019 25814263 PMC4437855

[3] Wang B Chen D. Evidence for seasonal mania: a review. J Psychiatr Pract. (2013) 19:301–8. 10.1097/01.pra.0000432600.32384.c5 23852105

[4] Rosenthal NE Sack DA Gillin JC Lewy AJ Goodwin FK Davenport Y. Seasonal affective disorder. A description of the syndrome and preliminary findings with light therapy. Arch Gen Psychiatry. (1984) 41:72–80. 10.1001/archpsyc.1984.01790120076010 6581756

[5] Maruani J Anderson G Etain B Lejoyeux M Bellivier F Geoffroy PA. The neurobiology of adaptation to seasons: relevance and correlations in bipolar disorders. Chronobiol Int. (2018) 35:1335–53. 10.1080/07420528.2018.1487975 29939763

[6] Berk M Kapczinski F Andreazza AC Dean OM Giorlando F Maes M. Pathways underlying neuroprogression in bipolar disorder: focus on inflammation, oxidative stress and neurotrophic factors. Neurosci Biobehav Rev. (2011) 35:804–17. 10.1016/j.neubiorev.2010.10.001 20934453

[7] Andreazza AC Young LT. The neurobiology of bipolar disorder: identifying targets for specific agents and synergies for combination treatment. Int J Neuropsychopharmacol. (2014) 17:1039–52. 10.1017/S1461145713000096 23449044

[8] Jeong H Dimick MK Sultan A Duong A Park SS El Soufi El Sabbagh D. Peripheral biomarkers of mitochondrial dysfunction in adolescents with bipolar disorder. J Psychiatr Res. (2020) 123:187–93. 10.1016/j.jpsychires.2020.02.009 32078836

[9] Wang JF Shao L Sun X Young LT. Increased oxidative stress in the anterior cingulate cortex of subjects with bipolar disorder and schizophrenia. Bipolar Disord. (2009) 11:523–9. 10.1111/j.1399-5618.2009.00717.x 19624391

[10] Andreazza AC Shao L Wang JF Young LT. Mitochondrial complex I activity and oxidative damage to mitochondrial proteins in the prefrontal cortex of patients with bipolar disorder. Arch Gen Psychiatry. (2010) 67:360–8. 10.1001/archgenpsychiatry.2010.22 20368511

[11] Versace A Andreazza AC Young LT Fournier JC Almeida JR Stiffler RS. Elevated serum measures of lipid peroxidation and abnormal prefrontal white matter in euthymic bipolar adults: toward peripheral biomarkers of bipolar disorder. Mol Psychiatry. (2014) 19:200–8. 10.1038/mp.2012.188 23358158 PMC3640681

[12] Cudney LE Sassi RB Behr GA Streiner DL Minuzzi L Moreira JC. Alterations in circadian rhythms are associated with increased lipid peroxidation in females with bipolar disorder. Int J Neuropsychopharmacol. (2014) 17:715–22. 10.1017/S1461145713001740 24438530

[13] Knorr U Simonsen AH Roos P Weimann A Henriksen T Christensen EM. Cerebrospinal fluid oxidative stress metabolites in patients with bipolar disorder and healthy controls: a longitudinal case-control study. Transl Psychiatry. (2019) 9:325. 10.1038/s41398-019-0664-6 31780642 PMC6882849

[14] Bartsiokas A Arsuaga J. Hibernation in hominins from Atapuerca, Spain half a million years ago. L'Anthropologie. (2020) 124:1–34. 10.1016/j.anthro.2020.102797

[15] Jasnow AM Huhman KL Bartness TJ Demas GE. Short-day increases in aggression are inversely related to circulating testosterone concentrations in male Siberian hamsters (Phodopus sungorus). Horm Behav. (2000) 38:102–10. 10.1006/hbeh.2000.1604 10964524

[16] Demas GE Polacek KM Durazzo A Jasnow AM. Adrenal hormones mediate melatonin-induced increases in aggression in male Siberian hamsters (Phodopus sungorus). Horm Behav. (2004) 46:582–91. 10.1016/j.yhbeh.2004.07.001 15555500

[17] Prendergast BJ Nelson RJ. Affective responses to changes in day length in Siberian hamsters (Phodopus sungorus). Psychoneuroendocrinology. (2005) 30:438–52. 10.1016/j.psyneuen.2004.08.008 15721056

[18] Scotti MA Belén J Jackson JE Demas GE. The role of androgens in the mediation of seasonal territorial aggression in male Siberian hamsters (Phodopus sungorus). Physiol Behav. (2008) 95:633–40. 10.1016/j.physbeh.2008.09.009 18824186

[19] van Rosmalen L van Dalum J Appenroth D Roodenrijs RTM de Wit L Hazlerigg DG. Mechanisms of temperature modulation in mammalian seasonal timing. FASEB J. (2021) 35:e21605. 10.1096/fj.202100162R 33913553

[20] Elmi A Zannoni A Govoni N Bertocchi M Forni M Ventrella D. Uncovering the physiological mechanisms underlying the roe deer (Capreolus capreolus) testicular cycle: Analyses of gelatinases and VEGF patterns and correlation with testes weight and testosterone. Animals (Basel). (2020) 10:1–13. 10.3390/ani10030444 32155893 PMC7143327

[21] Rattenborg NC Mandt BH Obermeyer WH Winsauer PJ Huber R Wikelski M. Migratory sleeplessness in the white-crowned sparrow (Zonotrichia leucophrys gambelii). PLoS Biol. (2004) 2:E212. 10.1371/journal.pbio.0020212 15252455 PMC449897

[22] Geoffroy PA Bellivier F Scott J Etain B. Seasonality and bipolar disorder: a systematic review, from admission rates to seasonality of symptoms. J Affect Disord. (2014) 168:210–23. 10.1016/j.jad.2014.07.002 25063960

[23] Akhter A Fiedorowicz JG Zhang T Potash JB Cavanaugh J Solomon DA. Seasonal variation of manic and depressive symptoms in bipolar disorder. Bipolar Disord. (2013) 15:377–84. 10.1111/bdi.12072 23621686 PMC3731411

[24] Watson-Whitmyre M Stetson MH. Reproductive refractoriness in hamsters: environmental and endocrine etiologies. In: Stetson MH editor. Processing of Environmental Information in Vertebrates. New York: Springer-Verlag. (1988). p. 219–249

[25] Reiter RJ Tan DX Manchester LC Paredes SD Mayo JC Sainz RM. Melatonin and reproduction revisited. Biol Reprod. (2009) 81:445–56. 10.1095/biolreprod.108.075655 19439728

[26] Maywood ES Bittman EL Hastings MH. Lesions of the melatonin- and androgen-responsive tissue of the dorsomedial nucleus of the hypothalamus block the gonadal response of male Syrian hamsters to programmed infusions of melatonin. Biol Reprod. (1996) 54:470–7. 10.1095/biolreprod54.2.470 8788201

[27] Leitner C Bartness TJ. An intact dorsomedial hypothalamic nucleus, but not the subzona incerta or reuniens nucleus, is necessary for short-day melatonin signal-induced responses in Siberian hamsters. Neuroendocrinology. (2011) 93:29–39. 10.1159/000320474 20847551 PMC3066241

[28] Jarjisian SG Piekarski DJ Place NJ Driscoll JR Paxton EG Kriegsfeld LJ. Dorsomedial hypothalamic lesions block Syrian hamster testicular regression in short day lengths without diminishing increased testosterone negative-feedback sensitivity. Biol Reprod. (2013) 89:23. 10.1095/biolreprod.113.109587 23782839 PMC4076360

[29] Jarjisian SG Butler MP Paul MJ Place NJ Prendergast BJ Kriegsfeld LJ. Dorsomedial hypothalamic lesions counteract decreases in locomotor activity in male Syrian hamsters transferred from long to short day lengths. J Biol Rhythms. (2015) 30:42–52. 10.1177/0748730414561546 25512303 PMC4379209

[30] DiMicco JA Samuels BC Zaretskaia MV Zaretsky DV. The dorsomedial hypothalamus and the response to stress: part renaissance, part revolution. Pharmacol Biochem Behav. (2002) 71:469–80. 10.1016/s0091-3057(01)00689-x 11830181

[31] Maywood ES Hastings MH. Lesions of the iodomelatonin-binding sites of the mediobasal hypothalamus spare the lactotropic, but block the gonadotropic response of male Syrian hamsters to short photoperiod and to melatonin. Endocrinology. (1995) 136:144–53. 10.1210/endo.136.1.7828525 7828525

[32] Raitiere MN Garyfallou VT Urbanski HF. Lesions in the anterior bed nucleus of the stria terminalis in Syrian hamsters block short-photoperiod-induced testicular regression. Biol Reprod. (1997) 57:796–806. 10.1095/biolreprod57.4.796 9314583

[33] Adidharma W Leach G Yan L. Orexinergic signaling mediates light-induced neuronal activation in the dorsal raphe nucleus. Neuroscience. (2012) 220:201–7. 10.1016/j.neuroscience.2012.06.020 22710065 PMC3412924

[34] Deats SP Adidharma W Lonstein JS Yan L. Attenuated orexinergic signaling underlies depression-like responses induced by daytime light deficiency. Neuroscience. (2014) 272:252–60. 10.1016/j.neuroscience.2014.04.069 24813431 PMC4090246

[35] Yan L Lonstein JS Nunez AA. Light as a modulator of emotion and cognition: Lessons learned from studying a diurnal rodent. Horm Behav. (2019) 111:78–86. 10.1016/j.yhbeh.2018.09.003 30244030 PMC6456444

[36] Rousseau K Atcha Z Loudon AS. Leptin and seasonal mammals. J Neuroendocrinol. (2003) 15:409–14. 10.1046/j.1365-2826.2003.01007.x 12622842

[37] Young CN Morgan DA Butler SD Mark AL Davisson RL. The brain subfornical organ mediates leptin-induced increases in renal sympathetic activity but not its metabolic effects. Hypertension. (2013) 61:737–44. 10.1161/HYPERTENSIONAHA.111.00405 23357182 PMC3832948

[38] Guerra MM González C Caprile T Jara M Vío K Muñoz RI. Understanding how the subcommissural organ and other periventricular secretory structures contribute via the cerebrospinal fluid to neurogenesis. Front Cell Neurosci. (2015) 9:480. 10.3389/fncel.2015.00480 26778959 PMC4689152

[39] Iliff JJ Wang M Liao Y Plogg BA Peng W Gundersen GA. A paravascular pathway facilitates CSF flow through the brain parenchyma and the clearance of interstitial solutes, including amyloid β. Sci Transl Med. (2012). 4:147ra111. 10.1126/scitranslmed.3003748 22896675 PMC3551275

[40] Jessen NA Munk AS Lundgaard I Nedergaard M. The glymphatic system: A beginner's guide. Neurochem Res. (2015) 40:2583–99. 10.1007/s11064-015-1581-6 25947369 PMC4636982

[41] Skipor J Thiery JC. The choroid plexus–cerebrospinal fluid system: undervaluated pathway of neuroendocrine signaling into the brain. Acta Neurobiol Exp (Wars). (2008). 68:414–28. Available online at: https://ane.pl/pdf/6843.pdf 18668165 10.55782/ane-2008-1708

[42] Prendergast BJ Gorman MR Zucker I. Establishment and persistence of photoperiodic memory in hamsters. Proc Natl Acad Sci U S A. (2000) 97:5586–91. 10.1073/pnas.100098597 10792054 PMC25872

[43] Liu B Burbach JP. Detection and high performance liquid chromatography identification of the summer rises of vasopressin and oxytocin immunoreactivity in the rat pineal gland. Endocrinology. (1987) 121:1716–20. 10.1210/endo-121-5-1716 3665843

[44] Duration of daylight for 2019 at Portland, Oregon, provided by US Naval Observatory. Available online at: https://aa.usno.navy.mil/data/docs/Dur_OneYear.php (accessed August 28, 2019)

[45] Teets NM Meuti ME. Hello darkness, my old friend: a tutorial of Nanda-Hamner protocols. J Biol Rhythms. (2021) 36:221–5. 10.1177/0748730421998469 33715479

[46] Elliott JA. Circadian rhythms, entrainment and photoperiodism in the Syrian hamster. p 203–17. In: Follett BK Follett DE editors. Biological Clocks in Seasonal Reproductive. Cycles Bristol: Wright. (1981).

[47] Mistlberger RE Antle MC. Entrainment of circadian clocks in mammals by arousal and food. Essays Biochem. (2011) 49:119–36. 10.1042/bse0490119 21819388

[48] Stevens JR Livermore A Jr. Eye blinking and rapid eye movement: pulsed photic stimulation of the brain. Exp Neurol. (1978) 60:541–56. 10.1016/0014-4886(78)90009-2 210034

[49] Stevens JR Livermore AA Jr. Fellman J. Loss of eye movements abolishes light entrainment of circadian mesolimbic catecholamine excitability: a function for REM? Life Sci. (1982) 30:495–501. 10.1016/0024-3205(82)90262-4 7200184

[50] Livermore AH Jr Stevens JR. Light transducer for the biological clock: a function for rapid eye movements. J Neural Transm. (1988) 72:37–42. 10.1007/bf01244630 3379386

[51] Benington JH Heller HC. REM-sleep timing is controlled homeostatically by accumulation of REM-sleep propensity in non-REM sleep. Am J Physiol. (1994) 266:R1992–2000. 10.1152/ajpregu.1994.266.6.R1992 8024056

[52] Khalsa SB Conroy DA Duffy JF Czeisler CA Dijk DJ. Sleep- and circadian-dependent modulation of REM density. J Sleep Res. (2002) 11:53–9. 10.1046/j.1365-2869.2002.00276.x 11869427

[53] Wehrle R Kaufmann C Wetter TC Holsboer F Auer DP Pollmächer T. Functional microstates within human REM sleep: first evidence from fMRI of a thalamocortical network specific for phasic REM periods. Eur J Neurosci. (2007) 25:863–71 17328781 10.1111/j.1460-9568.2007.05314.x

[54] Shea JL Mochizuki T Sagvaag V Aspevik T Bjorkum AA Datta S. Rapid eye movement (REM) sleep homeostatic regulatory processes in the rat: changes in the sleep-wake stages and electroencephalographic power spectra. Brain Res. (2008) 1213:48–56. 10.1016/j.brainres.2008.03.062 18455709 PMC2575066

[55] Fulda S Romanowski CP Becker A Wetter TC Kimura M Fenzel T. Rapid eye movements during sleep in mice: high trait-like stability qualifies rapid eye movement density for characterization of phenotypic variation in sleep patterns of rodents. BMC Neurosci. (2011) 12:110. 10.1186/1471-2202-12-110 22047102 PMC3228710

[56] Escudero M Márquez-Ruiz J. Tonic inhibition and ponto-geniculo-occipital-related activities shape abducens motoneuron discharge during REM sleep. J Physiol. (2008) 586:3479–91. 10.1113/jphysiol.2008.153254 18499728 PMC2538812

[57] Robinson J Bayliss SC Fielder AR. Transmission of light across the adult and neonatal eyelid in vivo. Vision Res. (1991) 31:1837–40. 10.1016/0042-6989(91)90031-y 1767502

[58] Figueiro MG. Individually tailored light intervention through closed eyelids to promote circadian alignment and sleep health. Sleep Health. (2015) 1:75–82. 10.1016/j.sleh.2014.12.009 26985450 PMC4790124

[59] Youngstrom TG Nunez AA. Comparative anatomy of the retino-hypothalamic tract in photoperiodic and non-photoperiodic rodents. Brain Res Bull. (1986) 17:485–92. 10.1016/0361-9230(86)90215-7 2430681

[60] Youngstrom TG Weiss ML Nunez AA. Retinofugal projections to the hypothalamus, anterior thalamus and basal forebrain in hamsters. Brain Res Bull. (1991) 26:403–11. 10.1016/0361-9230(91)90014-b 2049607

[61] Leak RK Moore RY. Identification of retinal ganglion cells projecting to the lateral hypothalamic area of the rat. Brain Res. (1997) 770:105–14. 10.1016/s0006-8993(97)00761-0 9372209

[62] Canteras NS Ribeiro-Barbosa ER Goto M Cipolla-Neto J Swanson LW. The retinohypothalamic tract: comparison of axonal projection patterns from four major targets. Brain Res Rev. (2011) 65:150–83. 10.1016/j.brainresrev.2010.09.006 20863850

[63] Delwig A Larsen DD Yasumura D Yang CF Shah NM Copenhagen DR. Retinofugal projections from melanopsin-expressing retinal ganglion cells revealed by intraocular injections of Cre-dependent virus. PLoS ONE. (2016) 11:e0149501. 10.1371/journal.pone.0149501 26895233 PMC4764510

[64] Thompson RH Swanson LW. Organization of inputs to the dorsomedial nucleus of the hypothalamus: a reexamination with Fluorogold and PHAL in the rat. Brain Res Brain Res Rev. (1998) 27:89–118. 10.1016/s0165-0173(98)00010-1 9622601

[65] Bowers RR Gettys TW Prpic V Harris RB Bartness TJ. Short photoperiod exposure increases adipocyte sensitivity to noradrenergic stimulation in Siberian hamsters. Am J Physiol Regul Integr Comp Physiol. (2005) 288:R1354–60. 10.1152/ajpregu.00792.2004 15821285

[66] Bartness TJ Liu Y Shrestha YB Ryu V. Neural innervation of white adipose tissue and the control of lipolysis. Front Neuroendocrinol. (2014) 35:473–93. 10.1016/j.yfrne.2014.04.001 24736043 PMC4175185

[67] Ryu V Zarebidaki E Albers HE Xue B Bartness TJ. Short photoperiod reverses obesity in Siberian hamsters via sympathetically induced lipolysis and Browning in adipose tissue. Physiol Behav. (2018) 190:11–20. 10.1016/j.physbeh.2017.07.011 28694154 PMC5988232

[68] Benarroch EE. The central autonomic network: functional organization, dysfunction, and perspective. Mayo Clin Proc. (1993) 68:988–1001. 10.1016/s0025-6196(12)62272-1 8412366

[69] Hadhazy A. Fact or Fiction: The Days (and Nights) Are Getting Longer. Scientific American. (2010). Available online at: https://www.scientificamerican.com/article/earth-rotation-summer-solstice/ (accessed 22 Nov, 2021).

[70] Gott JA Liley DT Hobson JA. Towards a functional understanding of PGO waves. Front Hum Neurosci. (2017) 11:89. 10.3389/fnhum.2017.00089 28316568 PMC5334507

[71] Nofzinger EA Mintun MA Wiseman M Kupfer DJ Moore RY. Forebrain activation in REM sleep: an FDG PET study. Brain Res. (1997) 770:192–201. 10.1016/s0006-8993(97)00807-x 9372219

[72] Datta S Siwek DF Patterson EH Cipolloni PB. Localization of pontine PGO wave generation sites and their anatomical projections in the rat. Synapse. (1998) 30:409–23. 10.1002/(SICI)1098-2396(199812)30:4<409::AID-SYN8>3.0.CO;2-# 9826233

[73] Luppi PH Clément O Fort P. Paradoxical (REM) sleep genesis by the brainstem is under hypothalamic control. Curr Opin Neurobiol. (2013) 23:786–92. 10.1016/j.conb.2013.02.006 23490549

[74] Jego S Glasgow SD Herrera CG Ekstrand M Reed SJ Boyce R. Optogenetic identification of a rapid eye movement sleep modulatory circuit in the hypothalamus. Nat Neurosci. (2013) 16:1637–43. 10.1038/nn.3522 24056699 PMC4974078

[75] Vetrivelan R Kong D Ferrari LL Arrigoni E Madara JC Bandaru SS. Melanin-concentrating hormone neurons specifically promote rapid eye movement sleep in mice. Neuroscience. (2016) 336:102–13. 10.1016/j.neuroscience.2016.08.046 27595887 PMC5056843

[76] Sitaram N Nurnberger JI Jr Gershon ES Gillin JC. Cholinergic regulation of mood and REM sleep: potential model and marker of vulnerability to affective disorder. Am J Psychiatry. (1982) 139:571–6. 10.1176/ajp.139.5.571 7072840

[77] Gold AK Sylvia LG. The role of sleep in bipolar disorder. Nat Sci Sleep. (2016) 8:207–14. 10.2147/NSS.S85754 eCollection (2016) 27418862 PMC4935164

[78] Zangani C Casetta C Saunders AS Donati F Maggioni E D'Agostino A. Sleep abnormalities across different clinical stages of bipolar disorder: A review of EEG studies. Neurosci Biobehav Rev. (2020) 118:247–57. 10.1016/j.neubiorev.2020.07.031 32738263

[79] Meek TH Nelson JT Matsen ME Dorfman MD Guyenet SJ Damian V. Functional identification of a neurocircuit regulating blood glucose. Proc Natl Acad Sci U S A. (2016) 113:E2073–82. 10.1073/pnas.1521160113 27001850 PMC4833243

[80] Raitiere MN. Does photoperiodism involve a seasonal and non-pathological Warburg effect? Med Hypotheses. (2020) 135:109447. 10.1016/j.mehy.2019.109447 31733532

[81] Bartness TJ Wade GN. Photoperiodic control of seasonal body weight cycles in hamsters. Neurosci Biobehav Rev. (1985) 9:599–612. 10.1016/0149-7634(85)90006-5 3909016

[82] Bélanger M Allaman I Magistretti PJ. Brain energy metabolism: focus on astrocyte-neuron metabolic cooperation. Cell Metab. (2011) 14:724–38. 10.1016/j.cmet.2011.08.016 22152301

[83] Verkhratsky A Matteoli M Parpura V Mothet JP Zorec R. Astrocytes as secretory cells of the central nervous system: idiosyncrasies of vesicular secretion. EMBO J. (2016) 35:239–57. 10.15252/embj.201592705 26758544 PMC4741299

[84] Zorec R Verkhratsky A Rodríguez JJ Parpura V. Astrocytic vesicles and gliotransmitters: Slowness of vesicular release and synaptobrevin2-laden vesicle nanoarchitecture. Neuroscience. (2016) 323:67–75. 10.1016/j.neuroscience.2015.02.033 25727638

[85] Silver R Bittman EL. Reproductive mechanisms: interaction of circadian and interval timing. Ann N Y Acad Sci. (1984) 423:488–514. 10.1111/j.1749-6632.1984.tb23455.x PubMed PMID: 6588810 6588810

[86] Horvat A Muhič M Smolič T Begić E Zorec R Kreft M. Ca2+ as the prime trigger of aerobic glycolysis in astrocytes. Cell Calcium. (2021) 95:102368. 10.1016/j.ceca.2021.102368 33621899

[87] Shibasaki T Sunaga Y Seino S. Integration of ATP, cAMP, and Ca2+ signals in insulin granule exocytosis. Diabetes. (2004) 53:S59–62. 10.2337/diabetes.53.suppl_3.s59 15561922

[88] Kilts CD Anderson CM. The simultaneous quantification of dopamine, norepinephrine and epinephrine in micropunched rat brain nuclei by on-line trace enrichment HPLC with electrochemical detection: Distribution of catecholamines in the limbic system. Neurochem Int. (1986) 9:437–45. 10.1016/0197-0186(86)90086-0 20493144

[89] Fatatis A Holtzclaw LA Avidor R Brenneman DE Russell JT. Vasoactive intestinal peptide increases intracellular calcium in astroglia: synergism with alpha-adrenergic receptors. Proc Natl Acad Sci U S A. (1994) 91:2036–40. 10.1073/pnas.91.6.2036 8134346 PMC43304

[90] Chneiweiss H Glowinski J Prémont J. Vasoactive intestinal polypeptide receptors linked to an adenylate cyclase, and their relationship with biogenic amine- and somatostatin-sensitive adenylate cyclases on central neuronal and glial cells in primary cultures. J Neurochem. (1985) 44:779–86. 10.1111/j.1471-4159.1985.tb12883.x 2857767

[91] Eiden LE Hökfelt T Brownstein MJ Palkovits M. Vasoactive intestinal polypeptide afferents to the bed nucleus of the stria terminalis in the rat: an immunohistochemical and biochemical study. Neuroscience. (1985) 15:999–1013. 10.1016/0306-4522(85)90249-0 3900807

[92] Gustafson EL Greengard P. Localization of DARPP-32 immunoreactive neurons in the bed nucleus of the stria terminalis and central nucleus of the amygdala: co-distribution with axons containing tyrosine hydroxylase, vasoactive intestinal polypeptide, and calcitonin gene-related peptide. Exp Brain Res. (1990) 79:447–58. 10.1007/bf00229315 1971224

[93] Kozicz T Vigh S Arimura A. Axon terminals containing PACAP- and VIP-immunoreactivity form synapses with CRF-immunoreactive neurons in the dorsolateral division of the bed nucleus of the stria terminalis in the rat. Brain Res. (1997) 767:109–19. 10.1016/s0006-8993(97)00737-3 9365022

[94] Dougalis AG Matthews GAC Bishop MW Brischoux F Kobayashi K Ungless MA. Functional properties of dopamine neurons and co-expression of vasoactive intestinal polypeptide in the dorsal raphe nucleus and ventro-lateral periaqueductal grey. Eur J Neurosci. (2012) 36:3322–32. 10.1111/j.1460-9568.2012.08255.x 22925150 PMC4273537

[95] Ahnaou A Yon L Arluison M Vaudry H Hannibal J Hamon M. Immunocytochemical distribution of VIP and PACAP in the rat brain stem: implications for REM sleep physiology. Ann N Y Acad Sci. (2006) 1070:135–42. 10.1196/annals.1317.095 16888155

[96] Lebow MA Chen A. Overshadowed by the amygdala: the bed nucleus of the stria terminalisemerges as key to psychiatric disorders. Mol Psychiatry. (2016) 21:450–63. 10.38/mp.2016.1 26878891 PMC4804181

[97] Hasue RH Shammah-Lagnado SJ. Origin of the dopaminergic innervation of the central extended amygdala and accumbens shell: a combined retrograde tracing and immunohistochemical study in the rat. J Comp Neurol. (2002) 454:15–33. 10.1002/cne.10420 12410615

[98] Meloni EG Gerety LP Knoll AT Cohen BM Carlezon WA Jr. Behavioral and anatomical interactions between dopamine and corticotropin-releasing factor in the rat. J Neurosci. (2006) 26:3855–63. 10.1523/JNEUROSCI.4957-05.2006 16597740 PMC6674129

[99] Carboni E Silvagni A Rolando MT Di Chiara G. Stimulation of in vivo dopamine transmission in the bed nucleus of stria terminalis by reinforcing drugs. J Neurosci. (2000). RC102. 10.1523/JNEUROSCI.20-20-j0002.2000 11027253 PMC6772858

[100] Vranjkovic O Pina M Kash TL Winder DG. The bed nucleus of the stria terminalis in drug-associated behavior and affect: A circuit-based perspective. Neuropharmacology. (2017). 122:100. 10.1016/j.neuropharm.2017.03.028 28351600 PMC5481847

[101] Maita I Bazer A Blackford JU Samuels BA. Functional anatomy of the bed nucleus of the stria terminalis-hypothalamus neural circuitry: implications for valence surveillance, addiction, feeding, and social behaviors. Handb Clin Neurol. (2021) 179:403–418. 10.1016/B978-0-12-819975-6.00026-1 34225978

[102] Aston-Jones G Delfs JM Druhan J Zhu Y. The bed nucleus of the stria terminalis. A target site for noradrenergic actions in opiate withdrawal. Ann N Y Acad Sci. (1999) 877:486–98. 10.1111/j.1749-6632.1999.tb09284.x 10415666

[103] Delfs JM Zhu Y Druhan JP Aston-Jones G. Noradrenaline in the ventral forebrain is critical for opiate withdrawal-induced aversion. Nature. (2000) 403:430–4. 10.1038/35000212 10667795

[104] Gaykema RP Goehler LE. Ascending caudal medullary catecholamine pathways drive sickness-induced deficits in exploratory behavior: brain substrates for fatigue?. Brain Behav Immun. (2011) 25:443–60. 10.1016/j.bbi.2010.11.005 21075199 PMC3039108

[105] Ashok AH Marques TR Jauhar S Nour MM Goodwin GM Young AH. The dopamine hypothesis of bipolar affective disorder: the state of the art and implications for treatment. Mol Psychiatry. (2017) 22:666–79. 10.1038/mp.2017.16 28289283 PMC5401767

[106] Lu J Jhou TC Saper CB. Identification of wake-active dopaminergic neurons in the ventral periaqueductal gray matter. J Neurosci. (2006) 26:193–202. 10.1523/JNEUROSCI.2244-05.2006 16399687 PMC6674316

[107] Hazlerigg DG Gonzalez-Brito A Lawson W Hastings MH Morgan PJ. Prolonged exposure to melatonin leads to time-dependent sensitization of adenylate cyclase and down-regulates melatonin receptors in pars tuberalis cells from ovine pituitary. Endocrinology. (1993) 132:285–92. 10.1210/endo.132.1.7678217 7678217

[108] Shen H Semba K. A direct retinal projection to the dorsal raphe nucleus in the rat. Brain Res. (1994) 635:159–68. 10.1016/0006-8993(94)91435-4 8173951

[109] Ren C Luan L Wui-Man Lau B Huang X Yang J Zhou Y. Direct retino-raphe projection alters serotonergic tone and affective behavior. Neuropsychopharmacology. (2013) 38:1163–75. 10.1038/npp.2013.35 23370156 PMC3656380

[110] Hu Z Mu Y Huang L Hu Y Chen Z Yang Y. A visual circuit related to the periaqueductal gray area for the antinociceptive effects of bright light treatment. Neuron. (2022). 1712–27. 10.1016/j.neuron.2022.02.009 35263618

[111] Trueta C De-Miguel FF. Extrasynaptic exocytosis and its mechanisms: a source of molecules mediating volume transmission in the nervous system. Front Physiol. (2012) 3:319. 10.3389/fphys.2012.00319 22969726 PMC3432928

[112] Borroto-Escuela DO Perez De La Mora M Manger P Narváez M Beggiato S. Brain dopamine transmission in health and parkinson's disease: modulation of synaptic transmission and plasticity through volume transmission and dopamine heteroreceptors. Front Synaptic Neurosci. (2018) 10:20. 10.3389/fnsyn.2018.00020 30042672 PMC6048293

[113] Vaarmann A Gandhi S Gourine AV Abramov AY. Novel pathway for an old neurotransmitter: dopamine-induced neuronal calcium signalling via receptor-independent mechanisms. Cell Calcium. (2010) 48:176–82. 10.1016/j.ceca.2010.08.008 20846720

[114] Berezhnov AV Fedotova EI Sergeev AI Teplov IY Abramov AY. Dopamine controls neuronal spontaneous calcium oscillations via astrocytic signal. Cell Calcium. (2021) 94:102359. 10.1016/j.ceca.2021.102359 33550209

[115] Müller MS Fox R Schousboe A Waagepetersen HS Bak LK. Astrocyte glycogenolysis is triggered by store-operated calcium entry and provides metabolic energy for cellular calcium homeostasis. Glia. (2014) 62:526–34. 10.1002/glia.22623 24464850

[116] Heimer L de Olmos J Alheid GF Záborszky L. Perestroika in the basal forebrain: opening the border between neurology and psychiatry. Prog Brain Res. (1991) 87:109–65. 10.1016/s0079-6123(08)63050-2 1866444

[117] Cai W Xue C Sakaguchi M Konishi M Shirazian A Ferris HA. Insulin regulates astrocyte gliotransmission and modulates behavior. J Clin Invest. (2018) 128:2914–26. 10.1172/JCI99366 29664737 PMC6025980

[118] Lance MS Miller SC Holtsclaw LI Turner BB. Photoperiod regulation of mineralocorticoid receptor mRNA expression in hamster hippocampus. Brain Research. (1997) 780:342–347. 9507186 10.1016/s0006-8993(97)01302-4

[119] Ronchi E Spencer RL Krey LC McEwen BS. Effects of photoperiod on brain corticosteroid receptors and the stress response in the golden hamster (Mesocricetus auratus). Brain Res. (1998) 780:348–51. 10.1016/s0006-8993(97)01303-6 9507189

[120] Pyter LM Adelson JD Nelson RJ. Short days increase hypothalamic-pituitary-adrenal axis responsiveness. Endocrinology. (2007) 148:3402–9. 10.1210/en.2006-1432 17395702

[121] Lemos DR Downs JL Raitiere MN Urbanski HF. Photoperiodic modulation of adrenal gland function in the rhesus macaque: effect on 24-h plasma cortisol and dehydroepiandrosterone sulfate rhythms and adrenal gland gene expression. J Endocrinol. (2009) 201:275–85. 10.1677/JOE-08-0437 19223397 PMC2746829

[122] Otsuka T Goto M Kawai M Togo Y Sato K Katoh K. Photoperiod regulates corticosterone rhythms by altered adrenal sensitivity via melatonin-independent mechanisms in Fischer 344 rats and C57BL/6J mice. PLoS ONE. (2012) 7:e39090. 10.1371/journal.pone.0039090 22720039 PMC3376106

[123] Kanikowska D Roszak M Rutkowski R Sato M Sikorska D Orzechowska Z. Seasonal differences in rhythmicity of salivary cortisol in healthy adults. J Appl Physiol. (1985) 126:764–770. 10.1152/japplphysiol.00972.2018 30702977

[124] Crestani CC Alves FH Gomes FV Resstel LB Correa FM Herman JP. Mechanisms in the bed nucleus of the stria terminalis involved in control of autonomic and neuroendocrine functions: a review. Curr Neuropharmacol. (2013) 11:141–59. 10.2174/1570159X11311020002 23997750 PMC3637669

[125] Johnson SB Emmons EB Anderson RM Glanz RM Romig-Martin SA Narayanan NS. A basal forebrain site coordinates the modulation of endocrine and behavioral stress responses via divergent neural pathways. J Neurosci. (2016) 36:8687–99. 10.1523/JNEUROSCI.1185-16.2016 27535914 PMC4987438

[126] Gungor NZ Paré D. Functional heterogeneity in the bed nucleus of the stria terminalis. J Neurosci. 36:8038–49. 10.1523/JNEUROSCI.0856-16.2016 27488624 PMC4971356

[127] Joseph-Vanderpool JR Rosenthal NE Chrousos GP Wehr TA Skwerer R Kasper S. Abnormal pituitary-adrenal responses to corticotropin-releasing hormone in patients with seasonal affective disorder: clinical and pathophysiological implications. J Clin Endocrinol Metab. (1991) 72:1382–7. 10.1210/jcem-72-6-1382 1851185

[128] Wehr TA Muscettola G Goodwin FK. Urinary 3-methoxy-4-hydroxyphenylglycol circadian rhythm. Early timing (phase-advance) in manic-depressives compared with normal subjects. Arch Gen Psychiatry. (1980) 37:257–63. 10.1001/archpsyc.1980.01780160027002 7362415

[129] Linkowski P Kerkhofs M Van Onderbergen A Hubain P Copinschi G L'Hermite-Balériaux M. The 24-hour profiles of cortisol, prolactin, and growth hormone secretion in mania. Arch Gen Psychiatry. (1994) 51:616–24. 10.1001/archpsyc.1994.03950080028004 8042910

[130] Swann AC Stokes PE Secunda SK Maas JW Bowden CL Berman N. Depressive mania vs. agitated depression: biogenic amine and hypothalamic-pituitary-adrenocortical function. Biol Psychiatry. (1994) 35:803–13. 10.1016/0006-3223(94)91143-6 7519061

[131] Valiengo LL Soeiro-de-Souza MG Marques AH Moreno DH Juruena MF Andreazza AC. Plasma cortisol in first episode drug-naïve mania: differential levels in euphoric vs. irritable mood. J Affect Disord. (2012) 138:149–52. 10.1016/j.jad.2011.11.046 22305430 PMC4479259

[132] Simard M Couldwell WT Zhang W Song H Liu S Cotrina ML. Glucocorticoids-potent modulators of astrocytic calcium signaling. Glia. (1999) 28:1–12. 10.1002/(sici)1098-1136(199910)28:1<1::aid-glia1>3.0.co;2-4 10498817

[133] Gasser PJ Lowry CA. Organic cation transporter 3: A cellular mechanism underlying rapid, non-genomic glucocorticoid regulation of monoaminergic neurotransmission, physiology, and behavior. Horm Behav. (2018) 104:173–82. 10.1016/j.yhbeh.2018.05.003 29738736 PMC7137088

[134] Gasser PJ Lowry CA Orchinik M. Corticosterone-sensitive monoamine transport in the rat dorsomedial hypothalamus: potential role for organic cation transporter 3 in stress-induced modulation of monoaminergic neurotransmission. J Neurosci. (2006) 26:8758–66. 10.1523/JNEUROSCI.0570-06.2006 16928864 PMC6674371

[135] Gasser PJ Orchinik M Raju I Lowry CA. Distribution of organic cation transporter 3, a corticosterone-sensitive monoamine transporter, in the rat brain. J Comp Neurol. (2009) 512:529–55. 10.1002/cne.21921 19025979

[136] Perry W Minassian A Paulus MP Young JW Kincaid MJ Ferguson EJ. reverse-translational study of dysfunctional exploration in psychiatric disorders: from mice to men. Arch Gen Psychiatry. (2009) 66:1072–80. 10.1001/archgenpsychiatry.2009.58 19805697 PMC2897252

[137] Young JW Goey AK Minassian A Perry W Paulus MP Geyer MA. The mania-like exploratory profile in genetic dopamine transporter mouse models is diminished in a familiar environment and reinstated by subthreshold psychostimulant administration. Pharmacol Biochem Behav. (2010) 96:7–15. 10.1016/j.pbb.2010.03.014 20363246 PMC2878916

[138] Young JW Cope ZA Romoli B Schrurs E Aniek Joosen van Enkhuizen J. Mice with reduced DAT levels recreate seasonal-induced switching between states in bipolar disorder. Neuropsychopharmacology. (2018) 43:1721–31. 10.1038/s41386-018-0031-y 29520059 PMC6006292

[139] Salvadore G Quiroz JA Machado-Vieira R Henter ID Manji HK Zarate CA Jr. The neurobiology of the switch process in bipolar disorder: a review. J Clin Psychiatry. (2010) 71:1488–501. 10.4088/JCP.09r05259gre 20492846 PMC3000635

[140] Judd LL Schettler PJ Brown ES Wolkowitz OM Sternberg EM Bender BG. Adverse consequences of glucocorticoid medication: psychological, cognitive, and behavioral effects. Am J Psychiatry. (2014) 171:1045–51. 10.1176/appi.ajp.2014.13091264 25272344

[141] Kawahara T Quinn MT Lambeth JD. Molecular evolution of the reactive oxygen-generating NADPH oxidase (Nox/Duox) family of enzymes. BMC Evol Biol. (2007) 7:109. 10.1186/1471-2148-7-109 17612411 PMC1940245

[142] Shimizu S Yamauchi Y Ishikawa A. Photoperiod following inoculation of arabidopsis with Pyricularia oryzae (syn. Magnaporthe oryzae) influences on the plant-pathogen interaction. Int J Mol Sci. (2021) 22. 10.3390/ijms22095004 34066846 PMC8125946

[143] Chen C Mahar R Merritt ME Denlinger DL Hahn DA. ROS and hypoxia signaling regulate periodic metabolic arousal during insect dormancy to coordinate glucose, amino acid, and lipid metabolism. Proc Natl Acad Sci U S A. (2021) 118. 10.1073/pnas.2017603118 33372159 PMC7817151

[144] Love NR Chen Y Ishibashi S Kritsiligkou P Lea R Koh Y. Amputation-induced reactive oxygen species are required for successful Xenopus tadpole tail regeneration. Nat Cell Biol. (2013) 15:222–8. 10.1038/ncb2659 23314862 PMC3728553

[145] Menon J Rozman R. Oxidative stress, tissue remodeling and regression during amphibian metamorphosis. Comp Biochem Physiol C Toxicol Pharmacol. (2007) 145:625–31. 10.1016/j.cbpc.2007.02.011 17395540

[146] Sousa KM Villaescusa JC Cajanek L Ondr JK Castelo-Branco G Hofstra W. Wnt2 regulates progenitor proliferation in the developing ventral midbrain. J Biol Chem. (2010) 285:7246–53. 10.1074/jbc.M109.079822 20018874 PMC2844173

[147] L'Episcopo F Tirolo C Testa N Caniglia S Morale MC Serapide MF. Wnt/β-catenin signaling is required to rescue midbrain dopaminergic progenitors and promote neurorepair in ageing mouse model of Parkinson's disease. Stem Cells. (2014). 32(8):2147-63. 10.1002/stem.1708 24648001 PMC4106883

[148] Wagenführ L Meyer AK Marrone L Storch A. Oxygen tension within the neurogenic niche regulates dopaminergic neurogenesis in the developing midbrain. Stem Cells Dev. (2016) 25:227–38. 10.1089/scd.2015.0214 26577812 PMC4742976

[149] Mesman S Smidt MP. Acquisition of the midbrain dopaminergic neuronal identity. Int J Mol Sci. (2020) 21. 10.3390/ijms21134638 32629812 PMC7369932

[150] Butruille L Vancamp P Demeneix BA Remaud S. Thyroid hormone regulation of adult neural stem cell fate: a comparative analysis between rodents and primates. Vitam Horm. (2021) 116:133–92. 10.1016/bs.vh.2021.02.009 33752817

[151] Helfer G Barrett P Morgan PJ. A unifying hypothesis for control of body weight and reproduction in seasonally breeding mammals. J Neuroendocrinol. (2019) 31:e12680. 10.1111/jne.12680 30585661

[152] Rambacher KM. Moniri NH. The β2-adrenergic receptor-ROS signaling axis: An overlooked component of β2AR function? Biochem Pharmacol. (2020) 171:113690. 10.1016/j.bcp.2019.113690 31697929 PMC6917825

[153] Huang YZ McNamara JO. Neuroprotective effects of reactive oxygen species mediated by BDNF-independent activation of TrkB. J Neurosci. (2012) 32:15521–32. 10.1523/JNEUROSCI.0755-12.2012 23115189 PMC3535312

[154] Juric DM Loncar D Carman-Krzan M. Noradrenergic stimulation of BDNF synthesis in astrocytes: mediation via alpha1- and beta1/beta2-adrenergic receptors. Neurochem Int. (2008) 52:297–306. 10.1016/j.neuint.2007.06.035 17681645

[155] Kilpatrick LE Hill SJ. Transactivation of G protein-coupled receptors (GPCRs) and receptor tyrosine kinases (RTKs): Recent insights using luminescence and fluorescence technologies. Curr Opin Endocr Metab Res. (2021) 16:102–12. 10.1016/j.coemr.2020.10.003 33748531 PMC7960640

[156] Conner JM Lauterborn JC Yan Q Gall CM Varon S. Distribution of brain-derived neurotrophic factor (BDNF) protein and mRNA in the normal adult rat CNS: evidence for anterograde axonal transport. J Neurosci. (1997) 17:2295–313 9065491 10.1523/JNEUROSCI.17-07-02295.1997 PMC6573520

[157] Hassani OK Rymar VV Nguyen KQ Huo L Cloutier JF Miller FD. The noradrenergic system is necessary for survival of vulnerable midbrain dopaminergic neurons: implications for development and Parkinson's disease. Neurobiol Aging. (2020) 85:22–37. 10.1016/j.neurobiolaging.2019.09.014 31734438

[158] Dulcis D Jamshidi P Leutgeb S Spitzer NC. Neurotransmitter switching in the adult brain regulates behavior. Science. (2013) 340:449–53. 10.1126/science.1234152 23620046

[159] Aumann TD Raabus M Tomas D Prijanto A Churilov L Spitzer NC. Differences in number of midbrain dopamine neurons associated with summer and winter photoperiods in humans. PLoS ONE. (2016) 11:e0158847. 10.1371/journal.pone.0158847 27428306 PMC4948786

[160] Petri I Diedrich V Wilson D Fernández-Calleja J Herwig A Steinlechner S. Orchestration of gene expression across the seasons: Hypothalamic gene expression in natural photoperiod throughout the year in the Siberian hamster. Sci Rep. (2016) 6:29689. 10.1038/srep29689 27406810 PMC4942572

[161] Hahn JD Swanson LW. Distinct patterns of neuronal inputs and outputs of the juxtaparaventricular and suprafornical regions of the lateral hypothalamic area in the male rat. Brain Res Rev. (2010) 64:14–103. 10.1016/j.brainresrev.2010.02.002 20170674 PMC2886810

[162] Dong HW Petrovich GD Watts AG Swanson LW. Basic organization of projections from the oval and fusiform nuclei of the bed nuclei of the stria terminalis in adult rat brain. J Comp Neurol. (2001) 436:430–55. 10.1002/cne.1079 11447588

[163] Groeger G Doonan F Cotter TG Donovan M. Reactive oxygen species regulate prosurvival ERK1/2 signaling and bFGF expression in gliosis within the retina. Invest Ophthalmol Vis Sci. (2012) 53:6645–54. 10.1167/iovs.12-10525 22956616

[164] Mossahebi-Mohammadi M Quan M Zhang JS Li X. FGF Signaling pathway: a key regulator of stem cell pluripotency. Front Cell Dev Biol. (2020) 8:79. 10.3389/fcell.2020.00079 32133359 PMC7040165

[165] Melvin RG Andrews MT. Torpor induction in mammals: recent discoveries fueling new ideas. Trends Endocrinol Metab. (2009) 20:490–8. 10.1016/j.tem.2009.09.005 19864159 PMC2788021

[166] Samms RJ Fowler MJ Cooper S Emmerson P Coskun T Adams AC. Photoperiodic regulation of FGF21 production in the Siberian hamster. Horm Behav. (2014) 66:180–5. 10.1016/j.yhbeh.2014.03.013 24909854

[167] Bienert GP Chaumont F. Aquaporin-facilitated transmembrane diffusion of hydrogen peroxide. Biochim Biophys Acta. (2014) 1840:1596–604. 10.1016/j.bbagen.2013.09.017 24060746

[168] Hallanger AE Wainer BH. Ascending projections from the pedunculopontine tegmental nucleus and the adjacent mesopontine tegmentum in the rat. J Comp Neurol. (1988) 274:483–515. 10.1002/cne.902740403 2464621

[169] Ruggiero DA Giuliano R Anwar M Stornetta R Reis DJ. Anatomical substrates of cholinergic-autonomic regulation in the rat. J Comp Neurol. (1990) 292:1–53. 10.1002/cne.902920102 2312784

[170] Hasselmo ME McGaughy J. High acetylcholine levels set circuit dynamics for attention and encoding and low acetylcholine levels set dynamics for consolidation. Prog Brain Res. (2004) 145:207–31. 10.1016/S0079-6123(03)45015-2 14650918

[171] Chiti Z Teschemacher AG. Exocytosis of norepinephrine at axon varicosities and neuronal cell bodies in the rat brain. FASEB J. (2007) 21:2540–50. 10.1096/fj.06-7342com 17405853

[172] Léger L Goutagny R Sapin E Salvert D Fort P Luppi PH. Noradrenergic neurons expressing Fos during waking and paradoxical sleep deprivation in the rat. J Chem Neuroanat. (2009) 37:149–57. 10.1016/j.jchemneu.2008.12.008 19152834

[173] Swanson LW Mogenson GJ Gerfen CR Robinson P. Evidence for a projection from the lateral preoptic area and substantia innominata to the 'mesencephalic locomotor region' in the rat. Brain Res. (1984) 295:161–78. 10.1016/0006-8993(84)90827-8 6201228

[174] Satoh K Fibiger HC. Cholinergic neurons of the laterodorsal tegmental nucleus: efferent and afferent connections. J Comp Neurol. (1986) 253:277–302. 10.1002/cne.902530302 2432101

[175] van Enkhuizen J Janowsky DS Olivier B Minassian A Perry W Young JW. The catecholaminergic-cholinergic balance hypothesis of bipolar disorder revisited. Eur J Pharmacol. (2015) 753:114–26. 10.1016/j.ejphar.2014.05.063 25107282 PMC4318788

[176] Cope ZA Lavadia ML Joosen AJM van de Cappelle CJA Lara JC Huval A. Converging evidence that short-active photoperiod increases acetylcholine signaling in the hippocampus. Cogn Affect Behav Neurosci. (2020) 20:1173–83. 10.3758/s13415-020-00824-2 32794101 PMC7718303

[177] Matthews L Berry A Ohanian V Ohanian J Garside H Ray D. Caveolin mediates rapid glucocorticoid effects and couples glucocorticoid action to the antiproliferative program. Mol Endocrinol. (2008) 22:1320–30. 10.1210/me.2007-0154 18308897 PMC5419537

[178] Nuñez FJ Johnstone TB Corpuz ML Kazarian AG Mohajer NN Tliba O. Glucocorticoids rapidly activate cAMP production via Gαs to initiate non-genomic signaling that contributes to one-third of their canonical genomic effects. FASEB J. (2020) 34:2882–95. 10.1096/fj.201902521R 31908022 PMC7027561

[179] Hasbi A Fan T Alijaniaram M Nguyen T Perreault ML O'Dowd BF. Calcium signaling cascade links dopamine D1-D2 receptor heteromer to striatal BDNF production and neuronal growth. Proc Natl Acad Sci U S A. (2009) 106:21377–82. 10.1073/pnas.0903676106 19948956 PMC2795506

[180] Troadec JD Marien M Darios F Hartmann A Ruberg M Colpaert F. Noradrenaline provides long-term protection to dopaminergic neurons by reducing oxidative stress. J Neurochem. (2001) 79:200–10. 10.1046/j.1471-4159.2001.00556.x 11595772

[181] Hanstein R Trotter J Behl C Clement AB. Increased connexin 43 expression as a potential mediator of the neuroprotective activity of the corticotropin-releasing hormone. Mol Endocrinol. (2009) 23:1479–93. 10.1210/me.2009-0022 19460861 PMC2737551

[182] Ishii T Warabi E Mann GE. Circadian control of BDNF-mediated Nrf2 activation in astrocytes protects dopaminergic neurons from ferroptosis. Free Radic Biol Med. (2019) 133:169–78. 10.1016/j.freeradbiomed.2018.09.002 30189266

[183] Schieber M Chandel NS. ROS function in redox signaling and oxidative stress. Curr Biol. (2014) 24:R453–62. 10.1016/j.cub.2014.03.034 24845678 PMC4055301

[184] Schirrmacher V. Less can be more: the hormesis theory of stress adaptation in the global biosphere and its implications. Biomedicines. (2021) 9. 10.3390/biomedicines9030293 33805626 PMC8000639

[185] Gerkema MP Davies WI Foster RG Menaker M Hut RA. The nocturnal bottleneck and the evolution of activity patterns in mammals. Proc Biol Sci. (2013) 280:20130508. 10.1098/rspb.2013.0508 23825205 PMC3712437

[186] Romeo S Viaggi C Di Camillo D Willis AW Lozzi L Rocchi C. Bright light exposure reduces TH-positive dopamine neurons: implications of light pollution in Parkinson's disease epidemiology. Sci Rep. (2013) 3:1395. 10.1038/srep01395 23462874 PMC3589725

[187] Russart KLG Nelson RJ. Light at night as an environmental endocrine disruptor. Physiol Behav. (2018) 190:82–9. 10.1016/j.physbeh.2017.08.029 28870443 PMC5839924

[188] Jing JN Wu ZT Li ML Wang YK Tan X Wang WZ. Constant light exerted detrimental cardiovascular effects through sympathetic hyperactivity in normal and heart failure rats. Front Neurosci. (2020) 14:248. 10.3389/fnins.2020.00248 32292327 PMC7124186

[189] Calkin CV. Insulin resistance takes center stage: a new paradigm in the progression of bipolar disorder. Ann Med. (2019) 51:281–93. 10.1080/07853890.2019.1659511 31453713 PMC7877881

[190] Kamintsky L Cairns KA Veksler R Bowen C Beyea SD Friedman A. Blood-brain barrier imaging as a potential biomarker for bipolar disorder progression. Neuroimage Clin. (2020) 26:102049. 10.1016/j.nicl.2019.102049 31718955 PMC7229352

[191] Buck MJ Squire TL Andrews MT. Coordinate expression of the PDK4 gene: a means of regulating fuel selection in a hibernating mammal. Physiol Genomics. (2002) 8:5–13. 10.1152/physiolgenomics.00076.2001 11842126

[192] Wu CW Biggar KK Storey KB. Biochemical adaptations of mammalian hibernation: exploring squirrels as a perspective model for naturally induced reversible insulin resistance. Braz J Med Biol Res. (2013) 46:1–13. 10.1590/1414-431x20122388 23314346 PMC3854349

[193] Martin SL. Mammalian hibernation: a naturally reversible model for insulin resistance in man? Diab Vasc Dis Res. (2008) 5:76–81. 10.3132/dvdr.2008.013 18537093

[194] Heni M Hennige AM Peter A Siegel-Axel D Ordelheide AM Krebs N. Insulin promotes glycogen storage and cell proliferation in primary human astrocytes. PLoS One. (2011) 6:e21594. 10.1371/journal.pone.0021594 21738722 PMC3124526

[195] Fernandez AM Hernandez E Guerrero-Gomez D Miranda-Vizuete A Torres Aleman I. A network of insulin peptides regulate glucose uptake by astrocytes: Potential new druggable targets for brain hypometabolism. Neuropharmacology. (2018) 136:216–222. 10.1016/j.neuropharm.2017.08.034 28859884

[196] Aalling NN Nedergaard M DiNuzzo M. Cerebral metabolic changes during sleep. Curr Neurol Neurosci Rep. (2018) 18:57. 10.1007/s11910-018-0868-9 30014344 PMC6688614

